# Dissection of metabolic reprogramming in polycystic kidney disease reveals coordinated rewiring of bioenergetic pathways

**DOI:** 10.1038/s42003-018-0200-x

**Published:** 2018-11-16

**Authors:** Christine Podrini, Isaline Rowe, Roberto Pagliarini, Ana S. H. Costa, Marco Chiaravalli, Ivano Di Meo, Hyunho Kim, Gianfranco Distefano, Valeria Tiranti, Feng Qian, Diego di Bernardo, Christian Frezza, Alessandra Boletta

**Affiliations:** 1 Division of Genetics and Cell Biology, San Raffaele Scientific Institute, Via Olgettina, 60, Milan, 20132 Italy; 2 INVEST- Marie Curie Postdoctoral Program, Università Vita-Salute San Raffaele, Via Olgettina, 60, Milan, 20132 Italy; 3 Telethon Institute of Genetics and Medicine, Pozzuoli, Naples, 80078 Italy; 4 MRC, Cancer Unit Cambridge, University of Cambridge, Hutchison/MRC Research Centre, Box 197, Cambridge Biomedical Campus, Cambridge, CB2 0XZ UK; 5 Medical Genetics and Neurogenetics Unit, Fondazione IRCCS Istituto Neurologico C. Besta, Via L. Temolo 4, 20126 Milan, Italy; 6 Division of Nephrology, Department of Medicine, University of Maryl and School of Medicine, Baltimore, MD 21201 USA; 7 Department of Chemical, Materials and Industrial Production, Engineering, University of Naples Federico II, 80125 Naples, Italy; 8 Present Address: Center for Medical Innovation, Seoul National University Hospital, Seoul, 03080 Korea

## Abstract

Autosomal Dominant Polycystic Kidney Disease (ADPKD) is a genetic disorder caused by loss-of-function mutations in *PKD1* or *PKD2*. Increased glycolysis is a prominent feature of the disease, but how it impacts on other metabolic pathways is unknown. Here, we present an analysis of mouse *Pkd1* mutant cells and kidneys to investigate the metabolic reprogramming of this pathology. We show that loss of *Pkd1* leads to profound metabolic changes that affect glycolysis, mitochondrial metabolism, and fatty acid synthesis (FAS). We find that *Pkd1*-mutant cells preferentially use glutamine to fuel the TCA cycle and to sustain FAS. Interfering with either glutamine uptake or FAS retards cell growth and survival. We also find that glutamine is diverted to asparagine via asparagine synthetase (ASNS). Transcriptional profiling of *PKD1*-mutant human kidneys confirmed these alterations. We find that silencing of *Asns* is lethal in *Pkd1*-mutant cells when combined with glucose deprivation, suggesting therapeutic approaches for ADPKD.

## Abstract (web summary)

Christine Podrini et al. present a comprehensive analysis of *Pkd1* mutant mouse cells and kidneys, providing new insight into autosomal dominant polycystic kidney disease (ADPKD). They find that *Pkd1* loss leads to profound metabolic changes, including asparagine synthase-driven glutamine anaplerosis.

## Introduction

Autosomal dominant Polycystic Kidney Disease (ADPKD) is a monogenic disorder, caused by loss-of-function mutations in either the *PKD1* (in ∼85% of cases) or *PKD2* (in the remaining ∼15%) genes^1–3^. The two proteins encoded by these genes, Polycystin-1 (PC-1) and Polycystin-2 (PC-2), are assembled into a functional complex at primary cilia, whose activity is defective in the disease. Additionally, PC-1 can be cleaved at several proteolytic sites^4^ resulting in products that can translocate either into the nucleus^5^, or into mitochondria^6^ or be localized at mitochondrial-associated membrane contacts^7,8^. Cysts are epithelial outpouches of clonal origin increasing in number and size along the life of affected individuals. Inheriting one mutant allele is not sufficient for cysts to arise, requiring a second event causing the function of the polycystins to drop below a critical threshold of activity^2^. Loss of heterozygosity has been reported in a subset of cysts suggesting that this might be one of the mechanisms^9^.

Together with the deregulation of several signalling cascades, ADPKD exhibits metabolic alterations^10–12^. Among these, defective glucose metabolism was shown to be a feature of the disease^11,12^ in a process resembling the Warburg effect observed in cancer. This finding prompted investigators to hypothesize that metabolic reprogramming might be a general feature of the disease^13,14^. Indeed, increased aerobic glycolysis, impaired beta-oxidation, reduced mitochondrial activity were reported in cellular and animal models lacking the *Pkd1* gene^6–8,11,15–19^, while altered glutamine usage was reported in a non-orthologous animal model of recessive polycystic kidney disease^20^. Likewise, inhibitors of glutamine usage proved effective in retarding disease progression in some, but not in other, models of the disease^21,22^. However, an overview of these metabolic alterations and their interconnections is still lacking. Metabolic profiling was carried out in non-orthologous models of the disease (i.e. cystogenesis caused by mutations in other genes)^23,24^, while a single study has attempted at profiling metabolites in the kidneys of a *Pkd1* orthologous mouse model^15^ reporting only a minimal metabolic change in murine kidneys derived from a ubiquitous, inducible inactivation of the *Pkd1* gene.

Here, we present a comprehensive metabolomics characterization of cells and renal tissues from a mouse model carrying the kidney-specific inactivation of the *Pkd1* gene. Our data indicate a broad metabolic rewiring that involves several pathways including central carbon metabolism and glutamine utilization. Finally, we show that glutamine metabolism is interlinked with asparagine synthesis in ADPKD and we identify the Asparagine Synthase (*Asns*) gene as an essential component of the process. Of note, targeting this enzyme by siRNA becomes lethal to ADPKD when associated with inhibition of glycolysis. Our data provide evidence of a broad and coordinated metabolic reprogramming in ADPKD, suggesting potential therapeutic strategies.

## Results

### Metabolomic profiling shows multiple changes in *Pkd1*-mutants

**Fig. 1:**
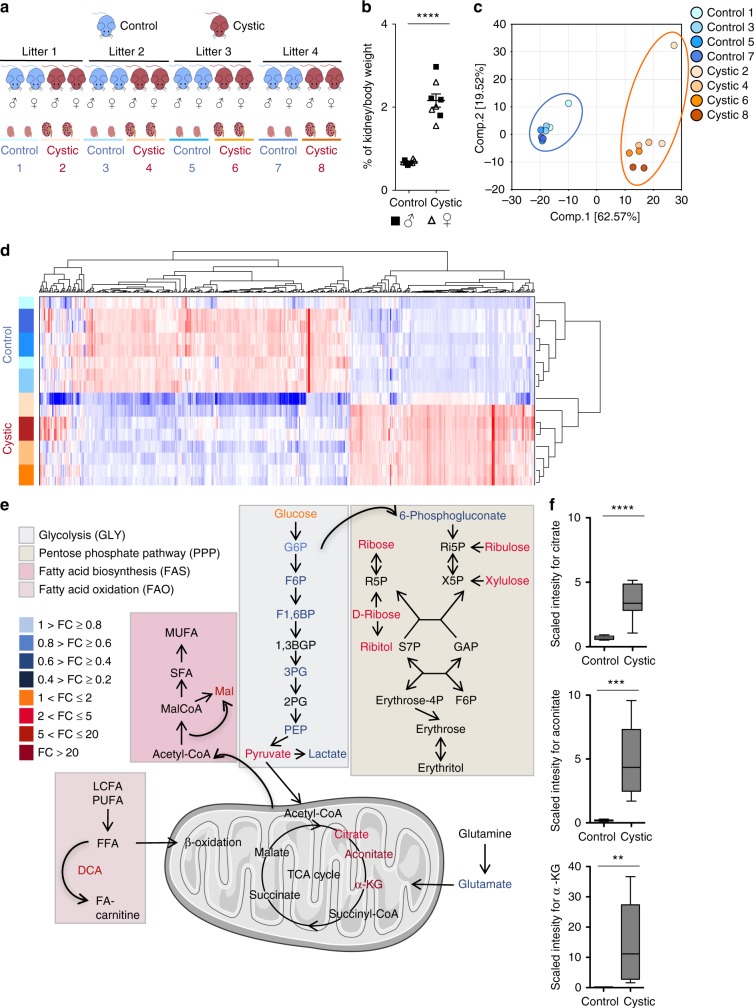
Global metabolomic profiling reveals defective TCA cycle in polycystic kidneys. **a** Study design of the experiment performed on kidneys from Ksp-cre;*Pkd1*^flox/−^ at P4. 4 litters each containing 2 cystic (red) Ksp-cre;*Pkd1*^flox/−^ and 2 control littermates Ksp-Cre;*Pkd1*^*flox/+*^ or *Pkd1*^*flox/+*^ (blue, used interchangeably) were collected. Samples were processed for analysis by Ultrahigh Performance Liquid Chromatography–Tandem Mass Spectrometry. **b** Dot plot view showing percentage of kidney/body weight in the cystic and control kidneys. **c** PCA applied to the identified metabolites, shows a good separation between cystic versus control kidneys. **d** Hierarchical clustering analysis shows good clustering between the groups of cystic and control kidneys samples. **e** Significant metabolites were colour-coded according to the pathway classification. Scheme of the Glycolysis (GLY), Pentose Phosphate Pathway (PPP), Tricarboxylic acid (TCA) cycle, Fatty acid biosynthesis (FAS), Fatty acids oxidation (FAO) in cystic versus control kidneys. Colour corresponds to the fold changes between cystic and control kidneys, orange-red are metabolites more abundant in cystic compared to the control kidneys, whereas blue labelled ones correspond to the less abundant metabolites in cystic kidneys compared to controls. The figure contains modified elements from Servier Medical Art (http://smart.servier.com/). All abbreviations are in Supplementary Table 2. **f** Box and whiskers view of the levels of TCA intermediates citrate, aconitate, and α-KG were assessed by LC-MS, they were significantly more abundant in cystic compared to the control kidneys. *n* = 8 independent biological replicates. Dot blots are shown as mean and error bars as SEM; Box and whiskers show median and 2.5-97.5 percentiles, n.s. not significant (*P* ≥ 0.05), *P < 0.05; **P < 0.01; ***P < 0.001; ****P < 0.0001, *t*-test for **b**, **e**, and **f**

To gather a comprehensive picture of the metabolic derangements observed in ADPKD mouse models, we applied non-targeted global metabolomics^25^ to an orthologous mouse model of ADPKD carrying inactivation of the *Pkd1* gene exclusively in the kidney as to avoid confounding effects derived from extra-renal inactivation. To this end we employed *KspCre;Pkd1*^*flox/−*^ kidneys carrying inactivation of the *Pkd1* gene in the distal tubules and collecting ducts of the kidney. To minimize phenotype variability in the experimental design we used a pure C57BL/6N background (i.e. >10 backcrosses) and performed the study upon precise timing of the day of birth of the animals (see methods). Furthermore, samples were collected at P4, when the kidneys are already cystic, but not yet functionally or structurally severely compromised (Supplementary Fig. 1a, b). Importantly, neither infiltration nor fibrosis could be detected at this time (Supplementary Fig. 1a). To further strengthen the outcome, we designed the study so that kidneys were collected from 4 litters containing each 2 cystic (*KspCre;Pkd1*^*flox/−*^*)* and 2 control littermates (*KspCre;Pkd1*^*flox/+*^ or *Pkd1*^*flox/+*^, used interchangeably) of each gender (8 males and 8 females in total) (Fig. 1a) to maximize the possibility to use intra-litter controls. Kidney-over-body weight ratios of *Pkd1*-mutant mice were significantly different from controls (*P* < 0.0001), but very similar in males and females at this neonatal stage (P4), possibly because sexual maturation is not yet achieved (Fig. 1b). Furthermore, no increase in *Epo* or *Vegf* transcription could be detected, thus excluding the possibility of these kidneys being hypoxic (Supplementary Fig. 1c). Application of Liquid Chromatography–Mass Spectrometry (LC-MS) resulted in the detection of 550 metabolites. A Principal Component Analysis (PCA) showed a clear separation between the cystic and control samples indicating a negligible influence of inter-gender and inter-litter differences in these samples (Fig. 1c). In line with this, hierarchical clustering analysis showed separation of the cystic and control samples (Fig. 1d). Paired *t*-test analysis was applied to take into account the intra-litter samples, resulting in the identification of 488 metabolites that significantly changed (adjusted *P* < 0.05), and 384 significantly different metabolites when considering both *P*-value and fold change (adjusted *P* < 0.05, absolute fold change >2) between cystic and control samples. A volcano plot of the data shows that 213 of these metabolites are downregulated, while 171 are upregulated, and that the main alterations are related to amino acids, carbohydrates, and lipids (Supplementary Fig. 1d and Supplementary Data 1).

A number of different metabolic pathways were found significantly altered (adjusted *P* < 0.05, absolute fold change > 2) (Supplementary Fig. 1e). Among these, striking differences were observed in metabolic pathways involved in bioenergetics, including glycolysis (GLY), pentose phosphate pathway (PPP), the tricarboxylic acid (TCA) cycle, fatty acid oxidation (FAO), and fatty acid biosynthesis (FAS) (Fig. 1e). The metabolites that appeared to be mostly accumulated in the cystic kidneys were citrate (*P* < 0.0001), aconitate (*P* = 0.0003), and α-ketoglutarate (α-KG) (*P* = 0.0079) (Fig. 1f). In line with this, KEGG-Pathways Based Enrichment Analysis showed that the TCA cycle is among the pathways affected with a statistically significant Enrichment Score (ES) (*P* ≤ 0.05) (Supplementary Fig. 1e).

These data indicate that the loss of *Pkd1* in the mouse kidney leads to profound metabolic changes, broader than what previously appreciated.

We then used a set of *Pkd1*^*+/+*^ and *Pkd1*^*−/−*^ Mouse Embryonic Fibroblasts (MEFs)^26^ to further investigate the metabolic changes in *Pkd1*-mutant cells. A metabolomic profiling revealed that *Pkd1*^*+/+*^ and *Pkd1*^*−/−*^ MEFs are well separated by PCA with many metabolites being significantly different between the two genotypes (adjusted *P* < 0.05) (Supplementary Fig. 2a, b) and that metabolic pathways such as glycolysis, PPP, FAS, and FAO were impaired (Supplementary Figure 2c and Supplementary Data 2). Likewise, targeted metabolomics profiling revealed an accumulation of the TCA cycle intermediates citrate, α-KG, succinate, and malate in *Pkd1*^*−/−*^ cells as compared to controls (Supplementary Fig. 2c, d). These results show that the response to *Pkd1* loss is strikingly similar between *Pkd1*-mutant MEFs and kidneys, supporting the validity of our cellular model for further mechanistic investigations.

**Fig. 2:**
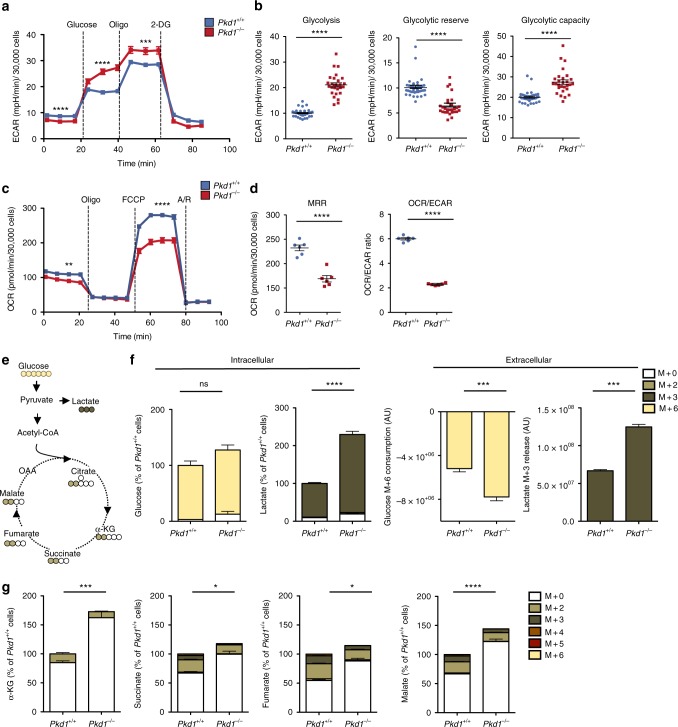
Impaired respiration and usage of glucose in the TCA cycle in *Pkd1*^*−/−*^ cells. **a**, **b** Representative analysis of ECAR outputs of *Pkd1*^*−/−*^ compared to *Pkd1*^*+/+*^ cells subject to glycolysis tests in response to glucose, oligomycin and 2-DG, **b** dot plots showing means with 28 to 39 replicate wells per group for glycolysis, glycolytic capaticity, and glycolytic reserve. Glycolysis was calculated on the 6th measurement time (after substraction of 3rd measurement); the glycolytic reserve was measured on the 9th measurement time (after substraction of 6th measurement)^;^ the glycolytic capacity was calculated on the 9th measurement time (after substraction of the 3rd measurement). **c**, **d** Representative analysis of OCR measurement in *Pkd1*^*+/+*^ and *Pkd1*^*−/−*^ cells in basal conditions and after sequential addition of oligomycin, FCCP and antimycin/rotenone (A/R). **d** Dot plots showing means with 6 replicate wells per group for: MRR calculated from the 10th measurement (after subtraction of the 13th measurement); OCR/ECAR ratio from basal measurement, 2nd time point. **a**–**d** Representative of three independent experiments. **e** The scheme illustrates the fate of glucose C atoms in glycolysis and Krebs cycle intermediates. Cells were incubated in glucose-free DMEM supplemented with 25 mM ^13^C_6_-glucose for 24 h. The uniformly labelled glucose (M + 6 yellow) leads to formation of M + 3 lactate (dark brown) or M + 2 TCA cycle intermediates after its first round (M + 2 light brown). **f** Percentage of isotopologue distribution of intracellular and extracellular glucose and lactate shows that glucose (M + 6) is more consumed and more converted into lactate (M + 3) in *Pkd1*^*−/−*^ cells in comparison to control cells (see Methods for calculations). **g** Percentage of isotopologue distribution of intracellular intermediates of the TCA: total α-KG, succinate, fumarate, and malate shows that the molecules coming from glucose (M + 2) are decreased in *Pkd1*^*−/−*^ MEFs compared to the control cells. Graphs (**f**, **g**) are means in percentages relative to control cells of six technical replicates from one experiment. Mean ± SEM were indicated, statistical significance is provided for total pool. n.s. not significant (*P* ≥ 0.05), *P < 0.05; **P < 0.01; ***P < 0.001; ****P < 0.0001, *t*-test

We first assessed whether the TCA cycle alterations could be associated with a defective cellular respiration in *Pkd1*^*−/−*^ cells as recently reported^7,15^. Consistent with this hypothesis, extracellular flux analysis of *Pkd1*^*−/−*^ MEFs showed enhanced glycolysis (*P* < 0.0001) (Fig. 2a, b) and defective respiration (Oxygen Consumption Rate, OCR) (*P* < 0.0001) (Fig. 2c, d) as compared to *Pkd1*^*+/+*^. Similar results on OCR were generated using primary *Pkd1*^*−/−*^ MEFs (Supplementary Fig. 3a) and murine Inner Medullary Collecting Duct Cells (mIMCD cells) where the *Pkd1* gene was silenced (*P* < 0.0001) (Supplementary Fig. 3b)^27^.

### Glucose entry into the TCA cycle is reduced in *Pkd1*^*−/−*^ cells

We then further investigate to what extent the loss of *Pkd1* affects central carbon metabolism. To track the contribution of glucose, one of the major carbon sources for the cells, to the TCA cycle in the *Pkd1*^*−/−*^ cells, we incubated cells with uniformly labelled ^13^C-glucose and followed the incorporation of ^13^C in downstream metabolites (Fig. 2e). Relative to control cells, *Pkd1*^*−/−*^ cells took up more glucose (*P* = 0.0002) and converted it into lactate (*P* = 0.0001), which is released into the culture medium (Fig. 2f and Supplementary Fig. 3c) in line with our previous findings^11,12^. Not surprisingly, the results showed that lactate almost entirely derives from glucose (Fig. 2f and Supplementary Fig. 3c). The data also confirmed that total α-KG  is significantly increased (*P* = 0.0002). Importantly, the data showed that the glucose-derived isotopologues of succinate (M + 2) (*P* = 0.0025), fumarate (M + 2) (*P* = 0.0001), and malate (M + 2) (*P* = 0.0023) were all significantly decreased in *Pkd1*^*−/−*^ MEFs, indicating a reduced contribution of glucose to the TCA cycle (Fig. 2g).

### Rewiring of glutamine metabolism in *Pkd1*^*−/−*^ cells

**Fig. 3:**
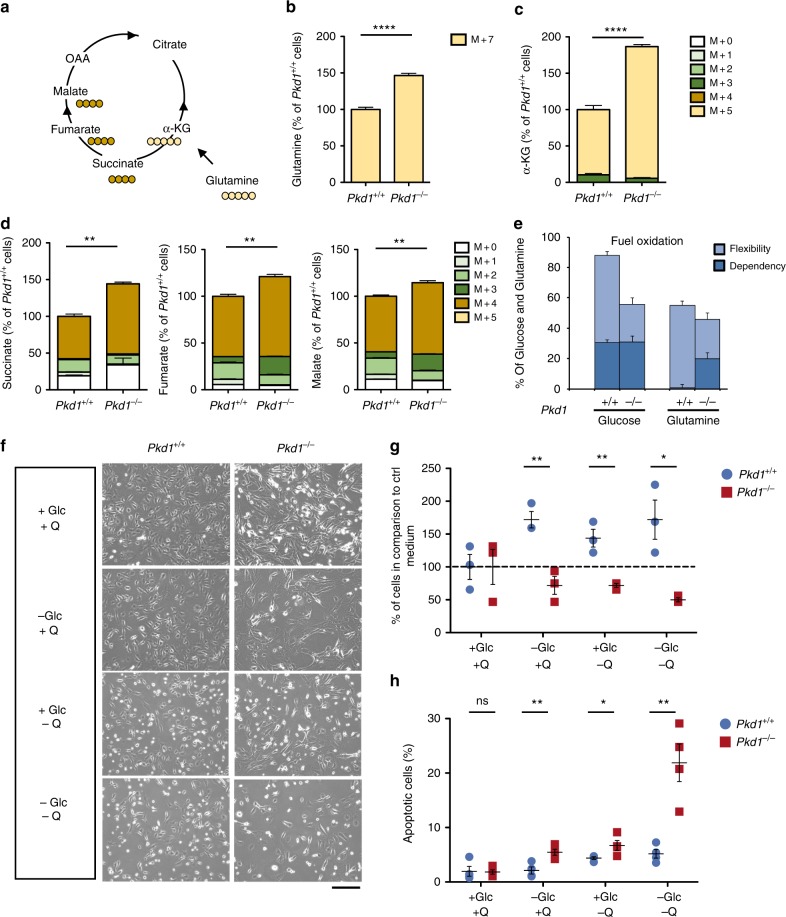
Metabolic rearrangement in bioenergetics pathways and glutaminolysis rewiring in *Pkd1*^*-/-*^ cells. **a** The scheme illustrates the fate of glutamine C atoms in Krebs cycle intermediates (Oxidative). Cells were incubated in glutamine-free DMEM supplemented with 4 mM ^13^C_5_–^15^N_2_ glutamine for 24 h. **b** Quantification of the intracellular labelled ^13^C_5_–^15^N_2_ glutamine (M+7) showing that *Pkd1*^*−/−*^ cells have a higher uptake compared to the controls. **c** Isotopologue distribution of intracellular α-KG shows that pools containing five ^13^C (M + 5), coming from labelled glutamine, were all significantly higher in *Pkd1*^*−/−*^ as compared with *Pkd1*^*+/+*^ cells. **d** Isotopologue distribution of intracellular succinate, fumarate, and malate shows that pools containing four ^13^C (M + 4), coming from labelled glutamine, were all significantly higher in *Pkd1*^*−/−*^ as compared with *Pkd1*^*+/+*^ cells. Graphs are means in percentages relative to control cells of six technical replicates from one experiment. statistical significance is provided for total pool. **e** Representative graph showing the results in percentage of the XFMitoFuel Flex Test analysis measuring OCR in response to either glucose or glutamine from at least seven technical replicates per well of two independent experiments. Data show a reduced oxidation of glucose in *Pkd1*^*−/−*^ cells and an increased dependency on glutamine compared to wild-type cells. **f** Cells were grown under starvation from either glucose or glutamine or both in 10% serum for 24 and 48 h after an overnight 0.5% serum. Representative images showing the cellular morphology of *Pkd1*^*−/−*^ compared to *Pkd1*^*+/+*^cells in starved and non-starved conditions. **g** Viability of *Pkd1*^*+/+*^ and *Pkd1*^*−/−*^ cells, expressed as percentage cell count compared to time 0, after 24 h of incubation in starvation from glucose, glutamine, or both. **h** Percentage of apoptotic cells assayed by TUNEL assay, showing significant higher percentage of apototic cells in starvation. Graphs (**f**–**h**) are representative of at least three independent experiments, data are means from at least three technical replicates. Mean ± SEM were indicated, n.s. not significant (*P* ≥ 0.05), *P < 0.05; **P < 0.01; ***P < 0.001; ****P < 0.0001. *t*-test (**b**, **c**, and **d**) and (**g** and **h**, comparison between each condition)

We next assessed the utilization of glutamine, another key carbon source for the cells. To this end, we incubated cells with ^13^C_5_-^15^N_2_-glutamine and the fate of glutamine-derived carbons and nitrogen was assessed by LC-MS (Fig. 3a). *Pkd1*^*−/−*^ cells exhibited increased glutamine uptake, compared to the controls (*P* < 0.0001) (Fig. 3b), and glutamine-derived (M + 5) α-KG (*P* < 0.0001) (Fig. 3c). In addition, *Pkd1*^*−/−*^ cells diverted glutamine towards the TCA cycle, as demonstrated by the increased levels of glutamine-derived succinate (M + 4) (*P* < 0.0001), fumarate (M + 4) (*P* < 0.0001), and malate (M + 4) (*P* < 0.0001) in *Pkd1*^*−/−*^ as compared with *Pkd1*^*+/+*^ cells (Fig. 3d). Next, we measured the dependency and flexibility of *Pkd1*^*−/−*^ cells relative to their controls (using the XF Mito Fuel Flex test, see Methods). This assay measures the OCR of *Pkd1*^*+/+*^ and *Pkd1*^*−/−*^ cells in the presence of glucose and glutamine followed by their blockade using specific inhibitors of the two metabolic pathways (2-Deoxy-d-glucose and BPTES, respectively). Data showed that *Pkd1*^*−/−*^ cells are dependent on glutamine for their OCR production (*P* = 0.0007), while the glucose-driven OCR is overall reduced (*P* < 0.0001) (Fig. 3e). These data suggest that cells lacking *Pkd1* increased their utilization of glutamine to fuel the TCA cycle and maintain their OCR, most likely as a compensatory mechanism due to the reduced funnelling of glucose into mitochondria. Based on these findings, we hypothesized that cells lacking functional *Pkd1* would become addicted to glutamine in addition to glucose. Indeed, starvation from either glutamine or glucose reduced cell numbers (*P* = 0.0068 *and P* = 0.0057, respectively) (Fig. 3f, g) and increased cell death in *Pkd1*^*−/−*^ cells (*P* = 0.047 *and P* = 0.0077, respectively) (Fig. 3h) relative to the controls. Importantly, starvation from both carbon sources had a synergistic effect both on cell number (*P* = 0.015) and on apoptosis (*P* = 0.0032) (Fig. 3h).

**Fig. 4:**
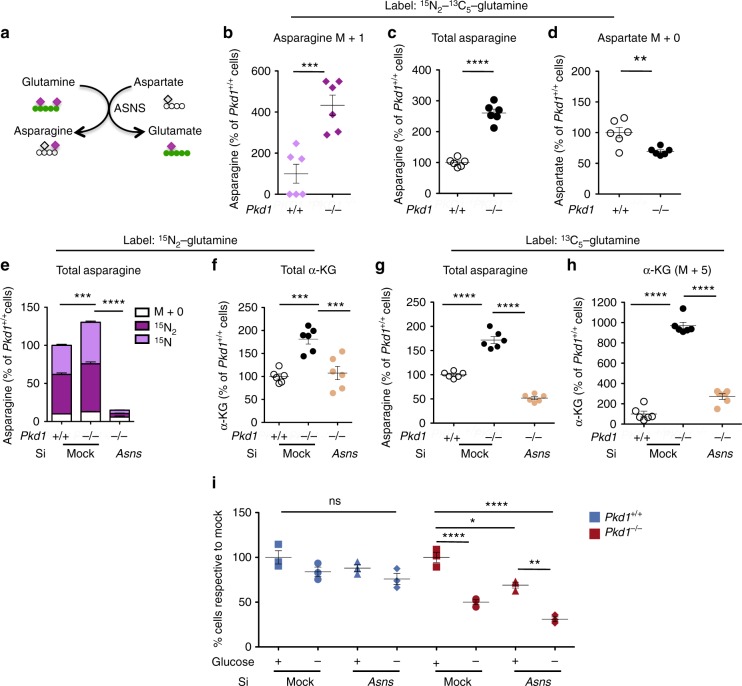
Glutamine usage is interlinked with asparagine synthase (ASNS) in ADPKD. **a** Schematic overview of the conversion of glutamine into glutamate by transamidating aspartate into asparagine by ASNS. **b** Labelled ^15^N-asparagine is significantly increased in the *Pkd1* mutant MEFs, **c** total levels of asparagine are increased compared to the control cells and **d** decreasing levels of aspartate (M + 0), expressed as percentage relative to controls. **e** Isotopologue distribution of intracellular asparagine showing that the pool coming from glutamine (^15^N_1_ and ^15^N_2_) is significantly decreased in si*Asns* compared to the mock *Pkd1*^*−/−*^ and control cells. **f** Total α-KG was significantly decreased in si*Asns* compared to the mock *Pkd1*^*−/−*^ and control cells in the ^15^N_2_ glutamine labelling. **g** Total asparagine was significantly decreased in si*Asns* compared to the mock *Pkd1*^*−/−*^ cells. **h** α-KG (M + 5) in the ^13^C_5_-glutamine labelling was significantly decreased in si*Asns* compared to the mock *Pkd1*^*−/−*^ and control cells. Data are means from six technical replicates from one experiment. **i** Representative graph showing the percentage of cell count compared to the respective mock controls. Silencing *Asns*, deprivation of glucose or both treatments in *Pkd1*^*+/+*^ resulted in no significant difference, whilsts each condition resulted in a significant reduction in cell number with an additive effect of *Asns* silencing with glucose starvation in *Pkd1*^*−/−*^ cells. Data are means from three technical replicates from two independent experiments. Mean ± SEM were indicated, n.s. not significant (*P* ≥ 0.05), *P < 0.05; **P < 0.01; ***P < 0.001; ****P < 0.0001, *t*-test for **b**, **c**, and **e**. ANOVA followed by Bonferroni for **e**, **f**, **g**, **h**, and **i**

Following the fate of the ^15^N_2_-labelled glutamine we noticed that there was a significant increase (*P* = 0.0006) of ^15^N-asparagine in the *Pkd1*^*−/−*^ cells suggesting an increased activity of asparagine synthase (Fig. 4a, b) and a concomitant increase of the overall levels of labelled asparagine derived from glutamine (*P* < 0.0001) (Fig. 4c), while aspartate was decreased in the same cells (*P* = 0.0060) (Fig. 4d). We therefore hypothesized that *Pkd1*^*−/−*^ cells exhibit increase asparagine synthesis from glutamine. Of note, quantitative RT-PCR revealed that asparagine synthase, the enzyme that generates asparagine from aspartate (Fig. 4a), was significantly upregulated in cells (*P* = 0.03) and murine kidneys (*P* = 0.0095) (Supplementary Fig. 4a). Furthermore, analysis of microarrays from murine and human samples confirmed a significant upregulation of this enzyme in both systems (*P* = 0.01 and *P* < 0.0001, respectively) (Supplementary Fig. 4a). Of interest, quantitative RT-PCR revealed no difference in the expression levels of the two enzymes *Gls* and *Glud1* (Supplementary Fig. 4b). To evaluate the relevance of asparagine biosynthesis, we silenced *Asns* in both *Pkd1*^*+/+*^ and in *Pkd1*^*−/−*^ cells (Fig. 4e–h and Supplementary Fig. 4c). As expected, the silencing of *Asns* reduced the intracellular levels of total asparagine in *Pkd1*^*−/−*^ cells (*P* < 0.0001)(Fig. 4e, g and Supplementary Fig. 4c). Furthermore, metabolic tracing with ^15^N_2_-glutamine showed that silencing of *Asns* indeed reduced the levels of labelled asparagine as expected (Fig. 4e) and importantly the total levels of α-KG (*P*  =0.0001) (Fig. 4f). Furthermore, metabolic tracing with ^13^C_5_-glutamine showed a significant reduction in the glutamine-derived α-KG (M + 5) (*P* < 0.0001) showing that *Asns* plays a central role in glutamine fuelling of the TCA cycle in *Pkd1*^*−/−*^ cells (Fig. 4h). Importantly, the downregulation of *Asns* also resulted in reduced cell numbers in *Pkd1*^*−/−*^ cells (*P* < 0.05), but not in controls (Fig. 4i). Furthermore, when *siAsns:Pkd1*^*−/−*^ cells were subject to glucose starvation we noticed a further decrease in cell numbers compared to controls treated in the same conditions (*P* < 0.0001) (Fig. 4i). We conclude that *Pkd1*^*−/−*^ cells depend on *Asns* for survival and when they are glucose deprived, downregulation of this enzyme further enhances their dependency.

Thus, our data show that increased glutaminolysis, interlinked with asparagine metabolism is an important feature of ADPKD and targeting *Asns* in conjunction with glycolysis might offer a novel therapeutic opportunity.

### Pkd1 loss leads to increased de novo fatty acid biosynthesis

**Fig. 5:**
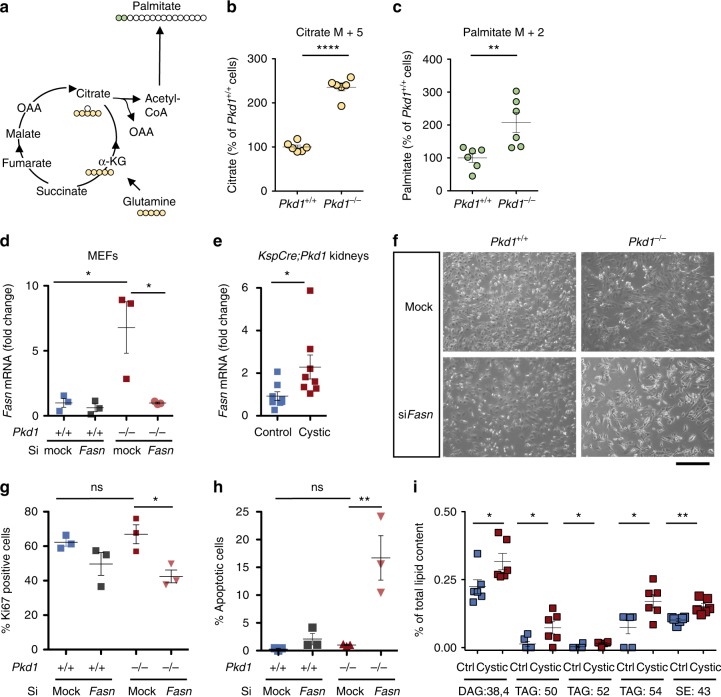
Glutaminolysis and fatty acids synthase dependence of *Pkd1*^*−/−*^ cells. **a** The scheme illustrates the fate of glutamine C atoms in TCA cycle intermediates and fatty acid biosynthesis. **b** Quantification of intracellular citrate (M + 5) labelled by glutamine was significantly higher in *Pkd1*^*−/−*^ as compared with *Pkd1*^*+/+*^ cells. **c** Incorporation of glutamine carbons into intracellular palmitate (M + 2) was significantly higher in *Pkd1*^*−/−*^ as compared with *Pkd1*^*+/+*^ cells. Data are means from six technical replicates from one experiment. **d** Dot plots showing means fold change, SEM, mRNA levels of *Fasn* in *Pkd1*^*−/−*^ compared to mock *Pkd1*^*+/+*^ cells and *SiFasnPkd1*^*−/−*^ compared to mock *Pkd1*^*−/*^^−^ normalized to *Hprt*. Three technical replicates of at least 3 independent experiments. **e** Dot plots showing means fold change, SEM, mRNA *Fasn* levels measured in control compared to cystic kidneys normalized to *Hprt*. *n* = 8, two technical replicates of at least two independent experiments. **f** Representative images showing cellular morphology of si*Fasn* in *Pkd1*^*+/+*^ and *Pkd1*^*−/−*^ and mock controls. **g** Representative graph of percentage Ki67-positive cells for mock and *SiFasn* for *Pkd1*^*+/+*^ and *Pkd1*^*−/−*^ cells after 48 h of transient silencing. Data from three technical replicates of at least two independent experiments. **h** Representative graphs showing percentage of apoptotic cells after 48 h of transfection, data from three technical replicates from three independent experiments. **i** Lipidomic profiling of cystic versus control kidneys showed altered percentage of diacylglycerol (DAG:38.4), three different species of triacylglycerol (TAG:50, TAG:52 TAG:54) and sterol esters (SE:43) from total lipid content, *n* = 6. Scale bar is 100 μm. Mean ± SEM were indicated, n.s. not significant (*p* ≥ 0.05), *P < 0.05; **P < 0.01; ***P < 0.001; ****P < 0.0001. *t*-test for **b**, **c**, ANOVA followed by Bonferroni for **d**, **e**, **g**, and **h** ANOVA for **i**

Of interest, the experiments of glutamine tracing also revealed that glutamine is used in an anaplerotic manner by *Pkd1*^*−/−*^ cells. Indeed, the increased level of citrate (M + 5) in the ^13^C_5_–^15^N_2_ glutamine labelling experiment (*P* < 0.0001) (Fig. 5a, b) suggested that glutamine undergoes reductive carboxylation in *Pkd1*^*−/−*^ cells. Given that reductive carboxylation has been linked with synthesis of lipogenic acetyl-CoA, we examined the labelling of palmitate, a fatty acid generated via de novo fatty acid biosynthesis. Consistent with this hypothesis, we noticed an increased labelling of glutamine-derived palmitate (M + 2) (Fig. 5c) in *Pkd1*^*−/−*^ cells compared to controls (*P* = 0.0094), although higher isotopologues could not be detected in this assay. Next, we tested whether *Pkd1*^*−/−*^ cells showed increased expression of fatty acids synthase (*Fasn*), a key enzyme involved in FAS. We found that *Fasn* is highly expressed in *Pkd1*^*−/−*^ cells (*P* = 0.044) and kidneys of *KspCre;Pkd1*^*flox/−*^ animals (*P* = 0.038) (Fig. 5d, e). Furthermore, its silencing reduced cell proliferation (*P* < 0.05) and enhanced cell apoptosis (*P* < 0.05) in *Pkd1*^*−/−*^ cells (Fig. 5f–h). In further support of an alteration in lipid metabolism, lipidomics profiling revealed a significant increase in diacylglycerols (DAG) (*P* = 0.03), triacyglycerols (TAG) (*P* = 0.03), and sterol esters (*P* = 0.002) in cystic kidneys compared to controls (Fig. 5i and Supplementary Data 3).

Overall, enhanced de novo fatty acids biosynthesis is a feature of ADPKD observed both in cells and in murine tissues and this process is necessary for *Pkd1*^*−/−*^ cells to proliferate and survive.

### A mathematical model reveals energetic pathways coordination

**Fig. 6:**
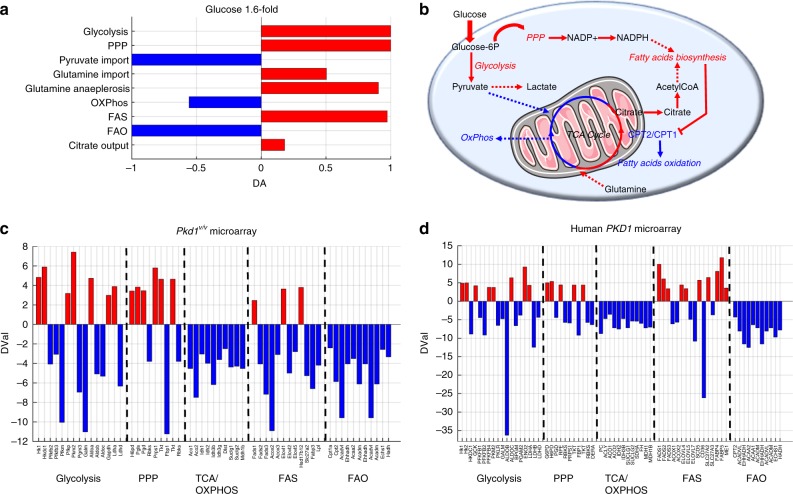
Global metabolic changes from in silico simulation and transcriptional profiling in polycystic kidney disease. **a** Analysis of metabolic rearrangement in bioenergetic pathways resulting from in silico simulations. The Differential Abundance (DA) score captures the average, gross changes of all metabolic fluxes in a pathway. A score of −1 indicates that all the simulated metabolic fluxes in the pathway decrease, while a score of 1 indicates that all in silico fluxes increase upon comparing the simulations of increased of glucose uptake with the computations of wild-type conditions. **b** Representation of the results of metabolic changes in the DFA in silico simulations upon increased glucose uptake. Data show that equilibrium is reached when upregulation of GLY, PPP, glutaminolysis, and FAS is achieved, while a reduction of OXPHOS and FAO. The figure contains modified elements from Servier Medical Art (http://smart.servier.com/). **c** Identification of genes differentially expressed in *Pkd1*^*V/V*^ kidneys versus controls in GLY, PPP, TCA/OXPHOS, FAS, and FAO. *D-val* represents the output of SAM algorithm launched with the parameter *delta* set to 1.0. All genes in the panel were differentially expressed between *Pkd1*^*V/V*^ animal model and wild-type model at P10 with a *FDR* equal to 0.1. Upregulated genes are shown in red, while downregulated ones are reported in blue. **d** Identification of genes differentially expressed in GLY, PPP, TCA/OXPHOS, FAS, and FAO in human *PKD1* microarrays data sets. *D-val* represents the output of SAM algorithm launched with the parameter *delta* set to 2.4. All genes in the panel were differentially expressed between cystic (small, medium, and large cysts) versus control tissues (minimal cystic and normal renal cortical tissues) with a *FDR* equal to zero. *n* = 4 for *Pkd1*^*V/V*^ animal model, *n* = 8 (normal and minimal cyst) and *n* = 13 for large, medium, small cyst for human *PKD1* microarrays

To gather a broader understanding of the metabolic changes observed, and to predict whether the different pathways are causally linked, we performed an in silico study. To this end we applied a recently described algorithm to predict changes in metabolic fluxes and metabolites across two conditions in a genome-scale metabolic model including 785 metabolites linked by 2589 enzymatic reactions (Differential Flux Balance Analysis, DFA)^28^. After removing all liver-specific functions, we used DFA to simulate in silico an increase in glucose uptake by imposing a constrain of enhanced glucose input driven by two transporters (TCDB:2.A.21.3.6 and TCDB:2.A.1.1.29, see Methods and Supplementary Data 4). We used as input the amount of increase in glucose uptake (1.6 fold) observed in the tracing experiments of *Pkd1*^*−/−*^ MEFs as compared to *Pkd1*^*+/+*^ controls (Fig. 2f). Next, we analysed the complete list of metabolites ranked according to their predicted change. This list was used as the input to KEGG-Pathways Based Enrichment Analysis, resulting in 31 pathways significantly enriched for altered metabolites (FDR ≤ 0.01). The most significant were glycolysis, TCA cycle, PPP, OXPHOS, and FAS (FDR ≤ 0.01) (Supplementary Fig. 5a). Importantly, this method allows to assign a direction to all the metabolic fluxes and to further predict additional alterations. Besides the metabolic pathways above we noticed a remarkable increase in glutamine uptake in the model system (DA_*p*_ = 0.5) (Fig. 6a, b and Supplementary Data 5), consistent with our glutamine labelling experiments. Further to this, the in silico simulations suggested that CPT1 and CPT2 metabolic fluxes might be reduced as a consequence of increased FAS. Indeed, qRT-PCR analysis showed that *Cpt1a* was significantly reduced in cells (*P* = 0.01) and kidneys lacking functional *Pkd1* (*P* = 0.009) (Supplementary Fig. 5b), further validating the predictions originated by the algorithm and in line with previous findings^15,16^.

The analysis of the in silico fluxes generated by the model revealed that most of the alterations in the discussed metabolic pathways is coordinated and likely causally linked, to the point that a single change in the increased uptake of glucose recapitulates a broad alteration in the other metabolic pathways (Fig. 6b).

### Analysis of ADPKD kidneys transcriptional profiles

We next reasoned that if these changes are indeed coordinated, they should occur in a synchronous fashion in the cystic kidneys. To address this point we first used a qPCR-based transcriptional profiling (qPCR arrays Qiagen^TM^) applied to a *KspCre;Pkd1*^*flox/−*^ kidneys analysed at the same time point in which metabolomics was performed (P4). Although the qPCR-based arrays provided a rather limited number of targets, the data showed a trend of alterations to key enzymes involved in GLY, PPP, FAS, and FAO, despite only a few displaying significant changes between the cystic and the control samples (*P* in the range 0.0002–0.03), (Supplementary Fig. 5c and Supplementary Data 6, 7).

To assess more comprehensively the expression of metabolic enzymes, we performed a microarray analysis on kidneys collected at P10 derived from a hypomorphic *Pkd1* mutant mouse (*Pkd1*^*V/V*^), which results in a milder polycystic kidney disease phenotype^29^. First, data were analysed by PCA and hierarchical clustering analysis, showing a very good separation of the samples in both assays (Supplementary Fig. 5d, e and Supplementary Data 8). Next, microarrays data have been analysed by means of the Significant Analysis of Microarrays (SAM) algorithm to identify differentially expressed genes^30^ followed by analysis of the full list of genes (Supplementary Data 9) involved in GLY, PPP, and FAS. These genes are mostly upregulated, while the genes involved in OXPHOS and FAO are markedly downregulated (*FDR* = 0.1) (Fig. 6c). Next, to validate our findings in human samples, we applied again the SAM algorithm^30^ to a previously published dataset of microarrays derived from the cystic kidneys of patients carrying *PKD1* mutations. Data were next screened for the complete list of genes involved in bioenergetics (Methods section) (Fig. 6d)^31^. The results showed that the metabolic alterations described in cellular and animal models of ADPKD (Figs. 1–3) and recapitulated by the mathematical model (Fig. 6a, b) and by the murine microarrays (Fig. 6c) are all perturbed in ADPKD1 kidneys including GLY, PPP, oxidative TCA cycle (TCA/OXPHOS), FAS, and FAO. Importantly, in human samples as well GLY, PPP, and FAS appear to be mostly upregulated, even if some isoforms of the enzymes are downregulated (*FDR* = 0). In contrast, both the OXPHOS and FAO enzymes are markedly downregulated (*FDR* = 0) (Fig. 6d).

These data taken together show that a general metabolic reprogramming of bioenergetic pathways is a hallmark of ADPKD and that likely most alterations are highly coordinated and occur simultaneously, opening unique opportunities for targeting them all with a few interventions.

## Discussion

In this study we performed a thorough analysis of the metabolic derangements observed in ADPKD using studies that range from global profiling in orthologous animal models to in silico flux analysis, in vitro carbon and nitrogen tracing, and finally validation in murine and human microarray data sets. The main conclusion of our studies is that a global metabolic reprogramming occurs in ADPKD, involving several pathways. Of all pathways involved, rewiring of central carbon metabolism is the most prominent and includes interlinked alterations of increased GLY, PPP, and FAS along with decreased OXPHOS and FAO. Of interest, we found that glutaminolysis is enhanced as a compensatory mechanism and used for both energy yielding purposes as well as for anabolic needs. Finally, we found that the usage of glutamine in ADPKD is interlinked with the asparagine metabolism and it involves upregulation of *Asns*. This is particularly intriguing because we found that targeting *Asns* is lethal in ADPKD cells, particularly when associated with glucose deprivation, thus opening new therapeutic perspectives for a combination therapy in this disease.

Based on previous studies it appeared that minimal changes in a few individual metabolic pathways might occur in ADPKD. Here we show instead that the metabolic alteration in ADPKD tissues is rather robust and occurs in multiple pathways. In addition to GLY and FAO, we find here alterations in the PPP, in FAO, in glutaminolysis and in OXPHOS. Furthermore, we show here that not only multiple metabolic pathways are occurring simultaneously, but that they are interconnected and likely causally linked.

A previous study has performed profiling of metabolites in an orthologous model of ADPKD, reporting only minimal changes in a few metabolites in *Pkd1*-mutant kidneys. The authors used a *Pkd1* orthologous mouse model carrying an inducible ubiquitous Cre^15^. Inactivation was induced after weaning, leading to a slowly progressive disease model with great variability in the renal phenotype^15^. This may explain why the metabolomics profiling data failed to separate in PCA based on the genotype. In the current study, we have used a kidney-specific Cre line to inactivate the *Pkd1* gene exclusively in the distal tubules and in collecting ducts (cadherin16-Cre, *KspCre*). This animal model develops a rapidly progressive phenotype with manifestation in the neonatal life. The different time points at which samples were analysed might in part explain the discrepancies with previous work^15^. More importantly, the animal model used in the current study shows reduced variability in the renal phenotype allowing for a good separation of the sample by PCA analysis and hierarchical clustering. Our data on the reduced FAO agree with those previously reported^15^, but we propose here that a much broader metabolic derangement than previously appreciated is present in ADPKD.

Importantly, we analysed renal samples at the cystic stage. Therefore, we cannot exclude that the metabolic alterations are secondary to cyst expansion. Relevant to this is the fact that recent investigations have unveiled an important correlation between chronic kidney disease (CKD) and metabolic changes^32^. Here we have excluded that at the time of analysis (P4) the animals are reaching CKD. Indeed, we observed a minimal initial increase in circulating urea, but absence of prominent inflammatory infiltrate or fibrosis. In addition, most of the metabolic alterations can be observed in isolated cells. Likewise, we have excluded that a prominent hypoxic state is present in the kidneys at the stages analysed. All these pieces of evidence suggest that the observed metabolic derangements are not secondary to CKD or hypoxia. However, based on the current knowledge we cannot exclude that, as disease progresses, additional alterations such as hypoxia and/or CKD can further contribute to disease worsening through additional metabolic stress.

In a recent elegant study, Hajarnis et al. has shown that the reduced FAO is secondary to the upregulation of microRNA-17, which in turn downregulates the expression levels of Pparα, ultimately reducing FAO^16^. Of interest, the authors were able to rescue the phenotype of *Pkd1* mutant mice by using fenofibrate, a natural compound acting as an agonist of Pparα and achieving enhancement of β-oxidation and OXPHOS^16,33^. Notably, the authors showed that the oncogene Myc is the main driver of miRNA17 expression^16^. It is important to note here that Myc is indeed considered a master regulator of metabolism in several types of cancer, with glycolysis and glutaminolysis both being regulated by this oncogene^34^.

In the current study we have used both LC-MS and GC-MS studies to perform metabolic profiling. While this type of analysis is informative, it also has the limitation that it provides a static snapshot of the metabolites detected, without a precise information on whether a given metabolic pathway is upregulated or downregulated. Indeed, we found decreased levels of most glycolytic intermediates in the static analysis of *KspCre;Pkd1*^*flox/−*^, including lactate. This is likely due to a rapid degradation or excretion of lactate in vivo. Indeed, by metabolic tracing with ^13^C_6_-glucose we previously showed detection of a large increase in lactate production in vivo^11,12^ when kidneys are collected after 40 minutes.

Metabolic tracing is a much more powerful tool to study the dynamic regulation of metabolic pathways. Our current studies show that *Pkd1*^*−/−*^ cells uptake large amounts of glucose used in the glycolytic pathway to generate lactate and that only minimal amounts of glucose are funnelled into the TCA cycle in mitochondria. Furthermore, we found that an increased uptake of glutamine is occurring in *Pkd1*^*−/−*^ cells as compared to the controls and it is oxidized into the TCA cycle, likely compensating for the reduced usage of glucose. Since the overall respiration of the cells is diminished, the likely explanation is that glutamine is used by these cells to preserve the mitochondrial membrane potential and avoid undergoing apoptosis. Indeed, in a previous study we demonstrated that the mitochondrial membrane potential was not majorly affected in the mutant cells^11^, despite the negligible contribution of glucose to mitochondria-generated ATP^11^. Our data are in disagreement with previous work reporting lack of enhanced glycolysis, and presence of defective mitochondrial membrane potential in *Pkd1*^*−/−*^ cells^6^. While differences in the cell type employed, in the immortalization procedure or in the culture conditions might account for part of the discrepancies, this cannot be the sole explanation for the controversial results. The glycolysis measurements in the previous studies are affected by wild-type cells being highly glycolytic, suggesting a minimal mitochondrial activity in the control cells and possibly limiting the capability to detect any increase in mutant cells^15^. Further studies will be required to reconcile the findings in the various different cell types analysed.

In the current study, we show that in addition to being oxidized in the TCA, glutamine is also used reductively in the *Pkd1*^*−/−*^ cells to generate citrate, which is transported into the cytosol and converted to acetyl-CoA, an essential substrate for fatty acids biosynthesis. The last process is upregulated in ADPKD likely to generate the membranes required for the proliferation of these cells. Indeed, silencing of *Fasn* greatly impacts cell proliferation and survival. Thus, our studies demonstrate a critical role for glutamine as a compensatory mechanism for the reduced usage of glucose in polycystic kidney disease. Our data provide the mechanistic explanation for two recent studies demonstrating that targeting glutaminolysis via the inhibition of the enzyme glutaminase (GLS) retards the progression of the disease. However, this effect was observed in some, but not in other animals carrying mutations in *Pkd1*^21,22^. Our study here provides a potential explanation for this discrepant observation. In our analyses we did not detect changes in the expression levels of GLS. This is in line with the results of Soomro et al., that showed no differences in vitro in the response of polycystic kidney disease or normal cells to inhibitors of GLS^22^. It should be noted, however, that despite this lack of increased expression we would expect that interfering with this enzyme should reduce glutamine uptake at least in vivo, because GLS is certainly the most commonly used enzyme to convert glutamine to glutamate in most tissues under physiological conditions. Indeed, treatment with the GLS inhibitors CB-839 resulted in a certain degree of reduction of cyst burden in two models of *Pkd1* mutants^21,22^, while it failed to improve the phenotype in a third one^22^. However, we have shown here that the utilization of glutamine in ADPKD is interlinked with the synthesis of asparagine via asparagine synthetase, ASNS, a transamidase that converts aspartate into asparagine while deaminating glutamine to form glutamate^35^. A recent study has shown that this reaction is used in physiological conditions by endothelial cells to utilize glutamine as a carbon source^36^. Our study shows that a similar mechanism is likely employed in ADPKD to consume glutamine. Indeed, silencing of *Asns* resulted in a complete rescue of the accumulation of α-KG, specifically by reducing the glutamine contribution to α-KG generation. Based on this, we propose that inhibiting ASNS would be a much more specific way to reduce glutamine usage in polycystic kidney disease, opening an important opportunity for a more targeted approach in ADPKD treatment. In line with this, we have found that silencing *Asns* impacts the growth of *Pkd1*^*−/−*^ cells and this is more prominent when cells are also deprived from glucose. The data indicates that indeed glutamine usage compensates for the lack of glucose utilization in the TCA cycle by the *Pkd1*^*−/−*^ cells and that targeting both processes at once is more effective than either one alone. In line with this, the starvation from both glucose and glutamine drastically enhances cell death in cells lacking *Pkd1*. Thus, inhibitors of *Asns* along with the glycolytic inhibitor 2-deoxy-d-glucose^11,12^ might offer a good therapeutic strategy in ADPKD and further studies should be devoted to test this possibility.

Ultimately, it should be noted that our results do not show the precise mechanism through which PC-1 regulates cellular and mitochondrial metabolism. Recent studies have reported a possible role for PC-1 in regulation of mitochondrial function either through regulation of mitochondrial Ca^2+^ uptake in mitochondrial-associated membranes^7^ or by direct translocation of a short fragment of the protein into the matrix of mitochondria^6^. Future efforts should be devoted to the understanding of the origin of the metabolic alterations in ADPKD, including the role of the Polycystins in regulation of mitochondrial function.

In conclusion, we report here the first broad overview of the metabolic derangement observed in ADPKD. Importantly, the altered pathways that we report in the current study expand our view on the potential use of inhibitors able to tackle the metabolic alterations to retard disease progression. The highly coordinated alterations observed offer a unique opportunity for targeting the process at multiple levels to block at once the capability of ADPKD cells to produce energy and to synthesize the building blocks needed for proliferation and survival.

## Methods

### Generation of *Pkd1*^*flox/−*^: Ksp-Cre mice

*Pkd1*^flox/−^: Ksp-Cre is a mouse model for ADPKD that develops massive enlarged kidney cysts within few days after birth and was generated by crossing *Pkd1*^*flox/flox*^ and *Pkd1*^+/−^:Ksp-Cre mice^37,38^. The age of the pups was accurately assessed by daily control of birth combined with the follow up of the variation of coat colours as described by Jackson Laboratories (https://www.jax.org). All mice used in these experiments were in a pure C57/BL6N genetic background (i.e. >10 backcrosses) and were maintained in specific pathogen free colonies handled by a service company provided at the San Raffaele Scientific Institute (Charles River). Mice received a sterilized (vacuum packed and irradiated) chow diet [25/18 CR, 5 % w/w crude fat (predominantly from soya products), soya oil 0.5% (14% kcal from fats, energy density of 2.64 kcal/g)]. All mice had ad libitum access to water and food. All experiments involving animals were performed under a protocol approved by an institutional ethical committee and, subsequently, by the Italian Ministry of Health (IACUC number: 736).

### Untargeted metabolomic analysis of kidneys and MEFs

The untargeted metabolomics in kidneys (Fig. 1) and MEFs (Supplementary Fig. 2) were carried out at Metabolon®. Briefly, samples were subjected to preparation and analysis as per the description of the supplier Metabolon®: to methanol extraction, split into aliquots for analysis by ultrahigh performance liquid chromatography/mass spectrometry (UPLC/MS). Thermo Scientific Q-Exactive high resolution/accurate mass spectrometer interfaced with a heated electrospray ionization (HESI-II) source and Orbitrap mass analyzer operated at 35,000 mass resolution. The sample extract was dried then reconstituted in solvents compatible to each of the four methods. Each reconstitution solvent contained a series of standards at fixed concentrations to ensure injection and chromatographic consistency. One aliquot was analysed using acidic positive ion conditions, chromatographically optimized for more hydrophilic compounds. In this method, the extract was gradient eluted from a C18 column (Waters UPLC BEH C18-2.1 × 100 mm, 1.7 µm) using water and methanol, containing 0.05% perfluoropentanoic acid (PFPA) and 0.1% formic acid. Another aliquot was analysed using acidic positive ion conditions, however it was chromatographically optimized for more hydrophobic compounds. In this method, the extract was gradient eluted from the same afore mentioned C18 column using methanol, acetonitrile, water, 0.05% PFPA, and 0.01% formic acid and was operated at an overall higher organic content. A third aliquot was analysed using basic negative ion optimized conditions using a separate dedicated C18 column. The basic extracts were gradient eluted from the column using methanol and water, however with 6.5 mM Ammonium Bicarbonate at pH 8. The fourth aliquot was analysed via negative ionization following elution from a HILIC column (Waters UPLC BEH Amide 2.1×150 mm, 1.7 µm) using a gradient consisting of water and acetonitrile with 10 mM Ammonium Formate, pH 10.8. The mass-spectrophotometric analysis alternated between MS and data-dependent MS^n^ scans using dynamic exclusion. The scan range varied slighted between methods but covered 70–1000 *m/z*. Metabolite concentrations were determined by automated ion detection, manual visual curation and were analysed in-line using software developed by Metabolon®^39^.

### Lipid extraction for untargeted mass spectrometry profiling

Mass spectrometry-based lipid analysis was performed at Lipotype GmbH (Dresden, Germany) as previously described and lipids were extracted using a two-step chloroform/methanol procedure^40^. Briefly, samples were treated as per the description of the company Lypotype GmbH: samples were spiked with internal lipid standard mixture containing: cardiolipin16:1/15:0/15:0/15:0 (CL), ceramide 18:1;2/17:0 (Cer), diacylglycerol 17:0/17:0 (DAG), hexosylceramide18:1;2/12:0 (HexCer), lyso-phosphatidate 17:0 (LPA), lyso-phosphatidylcholine 12:0 (LPC), lysophosphatidylethanolamine 17:1 (LPE), lyso-phosphatidylglycerol 17:1 (LPG), lyso-phosphatidylinositol 17:1 (LPI), lyso-phosphatidylserine 17:1 (LPS), phosphatidate 17:0/17:0 (PA), phosphatidylcholine 17:0/17:0 (PC), phosphatidylethanolamine 17:0/17:0 (PE), phosphatidylglycerol 17:0/17:0 (PG), phosphatidylinositol 16:0/16:0 (PI), phosphatidylserine 17:0/17:0 (PS), cholesterol ester 20:0 (CE), sphingomyelin 18:1;2/12:0;0 (SM), triacylglycerol 17:0/17:0/17:0 (TAG), and cholesterol D6 (Chol). After extraction, the organic phase was transferred to an infusion plate and dried in a speed vacuum concentrator. First step dry extract was re-suspended in 7.5 mM ammonium acetate in chloroform/methanol/propanol (1:2:4,V:V:V) and second step dry extract in 33% ethanol solution of methylamine in chloroform/methanol (0.003:5:1; V:V:V). All liquid handling steps were performed using Hamilton Robotics STARlet robotic platform with the Anti Droplet Control feature for organic solvents pipetting.

### Targeted metabolomics and stable isotope tracer analysis

Immortalized *Pkd1*^*+/+*^ and *Pkd1*^*−/−*^ MEFs^26^ and si*Asns Pkd1*^*−/−*^ were plated at 150,000 cells/well onto a 6-well plate (*n* = 6) and cultured in standard conditions. 24 h before sampling for ^13^C-labelling experiments, MEFs were washed twice with PBS and supplemented with media (DMEM (Gibco) supplemented with 10% dialysed foetal bovine serum (Gibco), 1% Penicillin Streptomycin (Pen/Strep, Gibco), Sodium Bicarbonate (3.7 g/l; Sigma Aldrich) containing either uniformly labelled ^13^C_6_-glucose (25 mM) (Cortecnet), ^13^C_5_-^15^N_2_-glutamine (4 mM) (Cortecnet), ^15^N_2_-Glutamine (Cambridge Isotope Laboratories), or ^13^C_5_-Glutamine (Cambridge Isotope Laboratories). Metabolites were extracted from cell pellets (intracellular) with 1 ml of extraction solution (methanol for highly pure liquid chromatography (Sigma Aldrich): acetonitrile gradient grade for liquid chromatography (Merck): ultrapure water (Sigma Aldrich), 50:30:20 with 100 ng ml^−1^ of HEPES (Sigma Aldrich) per million cells. The cell culture medium (extracellular) extracts were prepared by adding 750 µl of extraction solution to 50 µl of centrifuged cell culture medium. Samples were incubated at 4 °C for 15 min at 700 r.p.m., before centrifugation at 13,000 r.p.m. The supernatant was transferred into autosampler vials and stored at −80 °C prior to analysis by LC-MS.

LC-MS analysis was performed using a Q Exactive Orbitrap mass spectrometer coupled to a Dionex U3000 UHPLC system (Thermo Fisher Scientific). The liquid chromatography system was fitted with a Sequant ZIC-pHILIC column (150×2.1 mm) and guard column (20×2.1 mm) from Merck Millipore and temperature maintained at 45 °C. The mobile phase was composed of 20 mM ammonium carbonate and 0.1% ammonium hydroxide in water, and acetonitrile. The flow rate was set at 200 µl min^−1^ with the gradient described previously^41^. To expand on the range of metabolites covered in the analysis, the sample extracts were then run on a ZIC-HILIC column (150 mm × 4.6 mm) fitted with a guard column (20 mm × 2.1 mm) (both Merck Millipore). The aqueous mobile phase solvent used was 0.1% formic acid in water, and the organic mobile phase was 0.1% formic acid in acetonitrile. The flow rate was set at 300 μl min^−1^ and the column oven set to 30 °C, as described previously^41^. The mass spectrometer was operated in full MS mode with polarity switching, and samples were randomized in order to avoid bias due to machine drift and processed blindly. The acquired spectra were analysed using XCalibur Qual Browser and XCalibur Quan Browser software (Thermo Fisher Scientific).

Mass isotopologue distribution of metabolites was determined by integration of the corresponding peaks, and correction for natural abundance was performed using the Polly^TM^ IsoCorrect tool from the cloud-based platform Elucidata (https://polly.elucidata.io).

Consumption and release of metabolites was assessed by subtracting each metabolite total pool in the fresh medium (incubated in the absence of cells) from the pool found in the spent medium samples. The resulting value was then adjusted to the amount of protein generated during the incubation of the cells with the labelled substrate. For this purpose, cells were seeded in parallel plates and protein content was determined by the Bradford method at 0 and 24 h post medium change. Percentage of intracellular pool from each isotopologue was calculated respective of the control (for each metabolite).

### Glycolysis and mitochondrial respiration assays

Cells were plated at a density of 20,000 or 30,000 cells per well in a 96-wells Seahorse cell culture microplates and incubated in a 5% CO_2_ incubator at 37 °C overnight. The following day, 1 h before the test, culture media was replaced with pH-adjusted (pH = 7.4 ± 0.1) bicarbonate-free DMEM (Agilent) with 10 mM glucose (Sigma Aldrich), 1 mM sodium pyruvate (Gibco), and 2 mM l-glutamine (Gibco) for Mito Flex Test (Agilent) and Mito Stress Test or with 2 mM l-glutamine only for Glycolysis Stress Test (Agilent). The plate was then incubated at 37 °C for 1 h in a non-CO_2_ incubator. For the Mito Fuel Flex test and Mito Stress Test, OCR were measured using the Seahorse XF Mito Fuel Flex Test Kit XF and Mito Stress Test Kit (Agilent). Extracellular Acidification Rate (ECAR) was measured using XF Glycolysis Stress Test Kit (Agilent) on an XFe96 Analyzer (Agilent) following the manufacturer’s instructions. Cell numbers were normalized using CyQuant (Thermo Fisher).

### RNA extraction and microarray

Kidneys were removed from wild-type (*n* = 4) and *Pkd1*^V/V^ (*n* = 4) mice at P10 and homogenized in PBS buffer using mini handheld homogenizer. The homogenate was centrifuged and supernatant was discarded. Total RNAs were extracted from the pellet using an RNeasy mini kit (Qiagen) and the quality of total RNA samples was verified by a 260/280 ratio in NanoDrop and agarose gel electrophoresis. Further sample processing for microarray analysis was performed by the Genomics Core of Cleveland Clinic’s Lerner Research Institute, following the facility’s protocols, and hybridized to Illumina’s MouseRef-8 v2.0 BeadChip expression arrays.

### Real-time PCR analysis

RNA was isolated from plated cells or snap-frozen kidneys using the RNAspin Mini kit (GE Healthcare). Total RNA was isolated using the RNA-Isol lysis reagent according to the manufacturer’s instructions. For reverse transcription of RNA, Oligo(dT)_15_ primers (Promega) and ImProm-II Reverse Transcriptase (Promega) were used. Quantitative real-time PCR analysis was performed on duplicate using SYBR Green I master mix (Roche) on LightCycler 480 Instrument (Roche). For primers sequence see supplementary methods, Supplementary Table 1.

### Transient knockdown of Fasn and Asns

For transient knockdown of si*Fasn* and si*Asns* 20 nM (Ambion) pre-designed mRNA with the target sequences: FASN 5′-GGGAUCAUAAAGAUAACUUtt-3′ and 5′-AAGUUAUCUUUAUGAUCCCtc ASNS 5′-GGCCCUUGUUUAAAGCCAUtt-3′ and 5′-AUGGUUUAAACAAGGGCCtg-3’ along with control scrambled siRNA (siCONTROL, Dharmacon), were used following the manufacturer’s instructions and transfection control. For siRNA transfection cells were seeded into a 6-well plate, 100,000/well in 10% FBS (Gibco) in DMEM (Gibco) without antibiotics. The transfections were performed two times over 2 days at a final concentration of 30 nM using Lipofectamine 3000® following the manufacturer’s protocol. Total RNA was prepared from the cells 72 h after the first transfection and qRT-PCR was performed.

### Apoptosis cell assay

Cell death detection kit (TUNEL, Promega) was performed according to the manufacturer instructions after transient knockdown of si*Fasn* or after 48 h of starvation experiments (glutamine, glucose starvation). The protocols and quantifications were previously optimized^11^.

### Growth curve of MEFs in glucose and glutamine starvation

Immortalized *Pkd1*^*+/+*^ and *Pkd1*^*−/−*^ MEFs were plated at a density of 150,000 cells/well in DMEM (Gibco) supplemented with 0.5% FBS (Gibco) and 1% Pen/Strep (Gibco). After 16 h, medium was changed to control medium (DMEM, Gibco), supplemented with 10% FBS (Gibco), 1% Pen/Strep (Gibco), sodium pyruvate (1 mM; Gibco), sodium bicarbonate (44 mM; Sigma Aldrich), 25 mM glucose (Sigma Aldrich), and 4 mM glutamine (Gibco); glucose starvation medium (DMEM supplemented with 1 mM Sodium pyruvate, 44 mM sodium bicarbonate, 10% FBS, 1% Pen/Strep and 4 mM glutamine); glutamine starvation medium (DMEM supplemented with 10% FBS, 1% Pen/Strep, 1 mM sodium pyruvate, 44 mM sodium bicarbonate, and 25 mM glucose) or glucose and glutamine starvation medium (DMEM supplemented with 10% FBS and 1% Pen/Strep, 1 mM sodium pyruvate, 44 mM sodium bicarbonate). After 24 and 48 h, cells were trypsinized and counted with an automated cell counter (Countess cell counter, Invitrogen). Pictures from each sample at both time points were taken with a white field microscope, using a ×10 objective.

### Analysis using the KEGG-pathways based enrichment analysis

Analysis of the untargeted metabolomics studies was performed by implementing a KEGG-Pathways Based Enrichment Analysis (PBEA) system based on a similar concept than the Gene Set Enrichment Analysis (GSEA)^42^ which tests whether compounds involved in predefined pathways occur towards the top or bottom of the ranked query list. The method identifies altered pathways of the Kyoto Encyclopaedia of Genes and Genomes (KEGG)^43^ using metabolite data. An enrichment score and a statistical significance (*P*-value) have been computed for each pathway having at least one metabolite captured in the list of metabolites ranked, in descending order, according to a specific metric. Particularly, a fold change was used to rank the metabolites deriving from non-targeted global metabolomics, while the Differential Flux balance Analysis algorithm was used to rank the compounds resulting from the in silico simulations. In order to avoid ambiguous identification and to obtain reliable results only metabolites having a KEGG-identifier have been considered. The list of ranked metabolites named as L, and S for the set of compounds of a particular pathway. Analysis is performed to test whether the elements of S are randomly distributed through L or primarily found at the top or bottom of the list. The enrichment score reflects the degree to which S is over-represented at the extremes of L, and it is computed by walking down L, increasing a running-sum statistic when a metabolite in S is found and decreasing it when a compound not in S is encountered. The obtained score corresponds to a weighted Kolmogorov-Smirnov-like statistic^43^ and represents the maximum deviation from zero associated with a random walk. The *P*-value associated with an enrichment score has been computed as the fraction of 1000 random permutations of the elements of L that are at least as extreme as the original enrichment score, that has been derived from non-permuted elements. A Matlab® implementation of the method is available upon request.

### Differential abundance score

For a particular pathway *P*, the Differential Abundance (DA_*p*_) score is defined as:

$${\rm{DA}}_P = \frac{{\mathop {\sum }\nolimits_{r \in P} u_r}}{{\mathop {\sum }\nolimits_{r \in P} \left| {u_r} \right|}}$$

where *u*_*r*_ is the delta of in silico flux of reaction *r* computed by DFA. This score captures the tendency for a pathway to be upregulated or downregulated and varies from −1 to 1. A score of −1 indicates that all the metabolic fluxes associated with reactions in pathway *P* decreased respect to wild-type, while a score of 1 indicates that all fluxes increased when comparing with the wild-type simulation.

### In silico modelling and simulation of increased glycolysis

For the in silico studies the Genome-Scale Metabolic Model described in Pagliarini et al. was applied^28^. The model comprises 785 metabolites and 2589 enzymatic and transport reactions in eight compartments. In order to have more physiological results, the following additional constrains have been imposed: citrate cannot move from cytosol to mitochondria; pyruvate cannot move from mitochondria to cytosol; cytosolic enzyme LDH can only convert pyruvate to lactate and not vice versa. Then, Differential Flux balance Analysis was applied^28^ to simulate either the wild-type condition or an increase of glucose entering the cytosol (GLY). DFA is based on solving a linear optimization problem across 442 metabolic objectives for both the wild-type model and the perturbed model. For the current study, the liver-specific metabolic functions were removed. The average flux carried by each reaction across the different metabolic objectives for each of the two models is computed. For each reaction, the difference of the average flux in the wild-type model minus its value in the modified model are then considered. These differential fluxes are then used to rank the reactions from the ones that change the most in the modified model to the ones that change the least or do not change at all. Metabolites are then ranked according to the sum of the absolute values of the differential fluxes. In order to simulate an increase of the glucose uptake the results of the ^13^C-glucose labelled experiments showing an increase of 1.6 fold change in the uptake of glucose has been used. Therefore, the fluxes through the reactions Glucose(s) + Na + (s) − > Glucose(c) + Na + (c) (TCDB:2.A.21.3.6) and Glucose(s) < = > Glucose(c) (TCDB:2.A.1.1.29) has been forced, to be equal or greater than a specific threshold set to the their average value obtained in the wild-type simulations increased of 60%.

### Analysis of murine and human microarray data

In order to infer differentially expressed genes in microarrays data from *Pkd1*^V/V^ animal model at P10, a Matlab® implementation the SAM algorithm with *delta* = 1.0 was applied.

For human microarrays, data from Song et al.^31^ have been considered. First, affymetrix probe sets have been collapsed to one gene level by using the maximum expression value of the probe set in each gene. Then, the SAM algorithm has been applied, with *delta* = 2.4, to identify differentially expressed genes between transcripts in renal cysts of different sizes, and minimal cystic tissue plus normal renal cortical samples. The Matlab® implementation of SAM can be found in (https://it.mathworks.com/matlabcentral/fileexchange/42346-significance-analysis-of-microarrays–sam–using-matlab). In both microarrays analysis, after the computation of all the differentially expressed genes, those belonging to glycolysis, pentose phosphate pathway, TCA cycle/OXPHOS, fatty acid synthesis, and fatty acid oxidation were identified (Supplementary Data 9).

### Statistical analysis

For statistical analysis the Prism 5, GraphPad Software, and Matlab® were used using statistical analysis tool. *t*-test was used for all analysis of two groups. ANOVA statistical analysis followed by Bonferroni’s multiple comparison test was performed in all analysis where more than two groups were present. The *P*-values for each condition are indicated in the legends.

### Images creation

Figures 1e, 6b, and Suppl Fig. 2C contain modified images from Servier Medical Art, licensed under a Creative Common Attribution 3.0 Generic License. http://smart.servier.com/. All other images were generated by the authors.

## Electronic supplementary material

Supplementary Information

Description of Supplementary Data

Supplementary Data 1

Supplementary Data 2

Supplementary Data 3

Supplementary Data 4

Supplementary Data 5

Supplementary Data 6

Supplementary Data 7

Supplementary Data 8

Supplementary Data 9

Supplementary Data 10

Supplementary Data 11

## Data Availability

All data are provided within the manuscript. Raw data and elaboration for figures preparation are contained in Supplementary data 10. The raw data of metabolic tracing experiments ara available in Supplementary Data 11 and deposited into the MetaboLights database (reference number MTBLS677). Raw data of microarrays from *Pkd1*^*v/v*^ mice are available at GEO (GSE121563).
