# Supplementary material for: Dissection of metabolic reprogramming in polycystic kidney disease reveals coordinated rewiring of bioenergetic pathways
Source: Commun Biol. 2018 Nov 16;1:194. doi: 10.1038/s42003-018-0200-x (PMC6240072; doi:10.1038/s42003-018-0200-x)
Supplement: Supplementary file 1 — Supplementary Information [file 42003_2018_200_MOESM1_ESM.pdf]

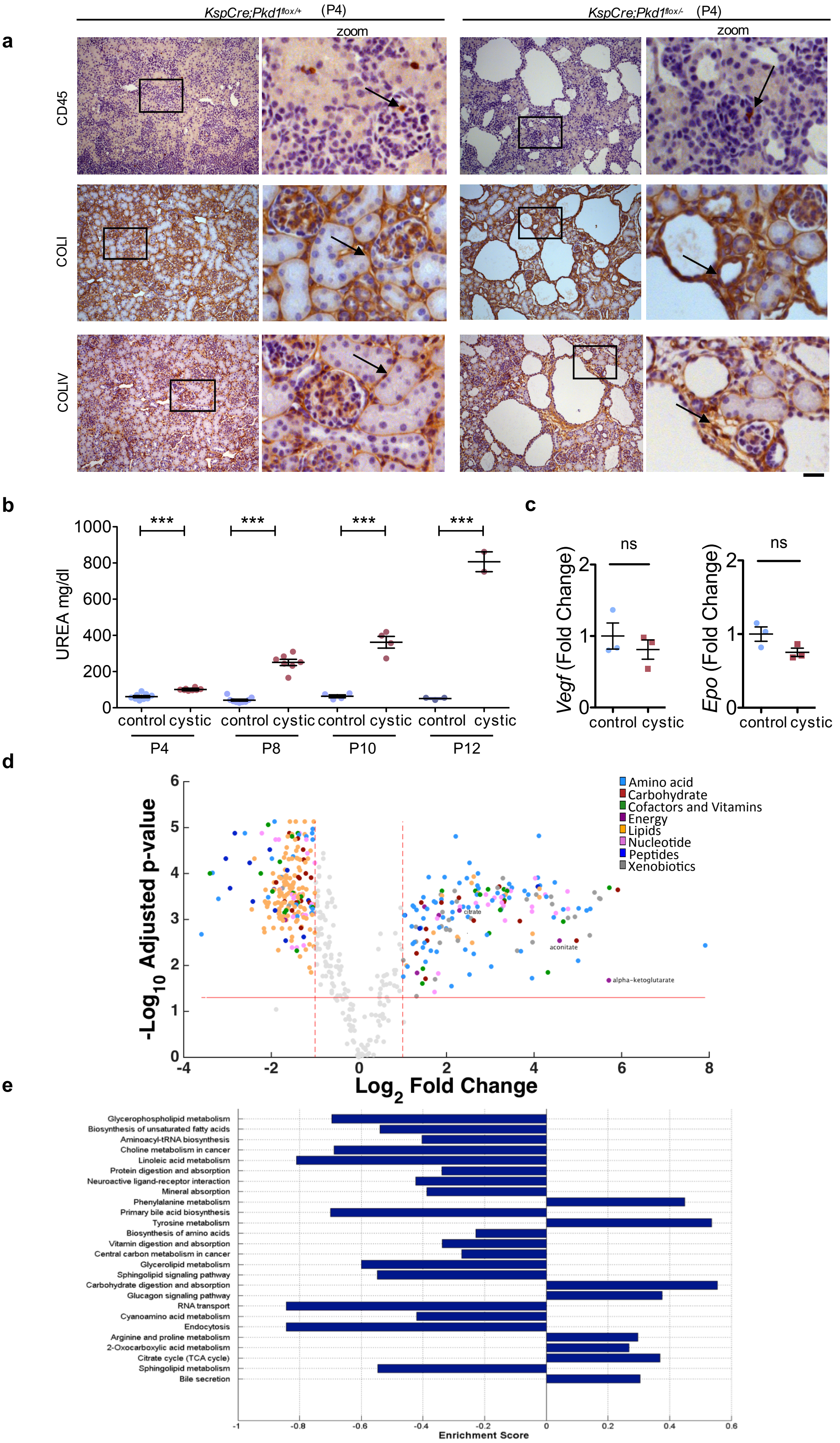
a
KspCre;Pkd1flox/+ (P4)
KspCre;Pkd1flox/- (P4)
zoom
zoom
CD45
COLI
COLIV
b
UREA mg/dl
1000
800
600
400
200
0
***
***
***
***
control cystic control cystic control cystic control cystic
P4
P8
P10
P12
c
Vegf (Fold Change)
Epo (Fold Change)
ns
ns
2
1
0
control cystic
control cystic
d
-Log10 Adjusted p-value
Log2 Fold Change
Amino acid
Carbohydrate
Cofactors and Vitamins
Energy
Lipids
Nucleotide
Peptides
Xenobiotics
citrate
aconitate
alpha-ketoglutarate
e
Glycerophospholipid metabolism
Biosynthesis of unsaturated fatty acids
Aminoacyl-tRNA biosynthesis
Choline metabolism in cancer
Linoleic acid metabolism
Protein digestion and absorption
Neuroactive ligand-receptor interaction
Mineral absorption
Phenylalanine metabolism
Primary bile acid biosynthesis
Tyrosine metabolism
Biosynthesis of amino acids
Vitamin digestion and absorption
Central carbon metabolism in cancer
Glycerolipid metabolism
Sphingolipid signaling pathway
Carbohydrate digestion and absorption
Glucagon signaling pathway
RNA transport
Cyanoamino acid metabolism
Endocytosis
Arginine and proline metabolism
2-Oxocarboxylic acid metabolism
Citrate cycle (TCA cycle)
Sphingolipid metabolism
Bile secretion
Enrichment Score

Supplementary figure 1. a Representative immunohistochemistry of cystic kidneys and control kidneys (P4) against CD45, Collagen I (COLI), Collagen IV (COLIV), respective markers of inflammation and fibrosis, showing no inflammation and only very mild fibrosis. Panels on the left and on the right show higher magnification. Bar represents 100μm. Immunohistochemistry are representative of two independent experiments. **b** Blood urea nitrogen (BUN) in mg/dL showing higher levels of BUN in cystic mice when compared to littermate controls collected at P4, P8, P10 and P12. Dot blot shown as mean standard error bars as SEM of two to nine independent biological replicates, **c** *Epo* and *Vegf* mRNA expression was assessed by quantitative RT-PCR, normalised to HPRT. No significant increase was detected for *Epo* and *Vegf* in the cystic kidneys when compared to the controls. Representative of three independent experiments. **d** Volcano plot of the 550 named metabolites profiled from mice kidneys. 384 metabolites exhibited significant changes (adjusted p value < 0.05, absolute fold change > 2) when comparing cystic kidneys with control kidneys. 171 metabolites exhibited a significant increase whilst 213 metabolites exhibited a significant decrease. Paired Student's *t*-tests were used to calculate statistical significance, and p values were corrected by using the Benjamini-Hochberg procedure (statistical analysis for all the 550 named metabolites can be found in **Supplementary Data 1**. Differentially abundant metabolites of different categories have been individually colour coded. **e** KEGG-Pathways Based Enrichment Analysis applied to the list of metabolites deriving from non-target global metabolomics performed on the *KspCre;Pkd1*$^{flox/-}$ kidneys and ranked in descending order according to the p values. It reveals 26 mouse pathways with a statistically significant ($p \leq 0.05$) enrichment score. Particularly, 8 pathways are enriched for upregulated metabolites whilst 18 are enriched for downregulated ones. Mean +/- SEM were indicated, n.s., not significant ($p \geq 0.05$), *p<0.05; **p<0.01;***p<0.001;****p<0.0001. *t*-test for **c** and ANOVA for **b**. Data in **d** and **e** were obtained from eight independent biological replicates.

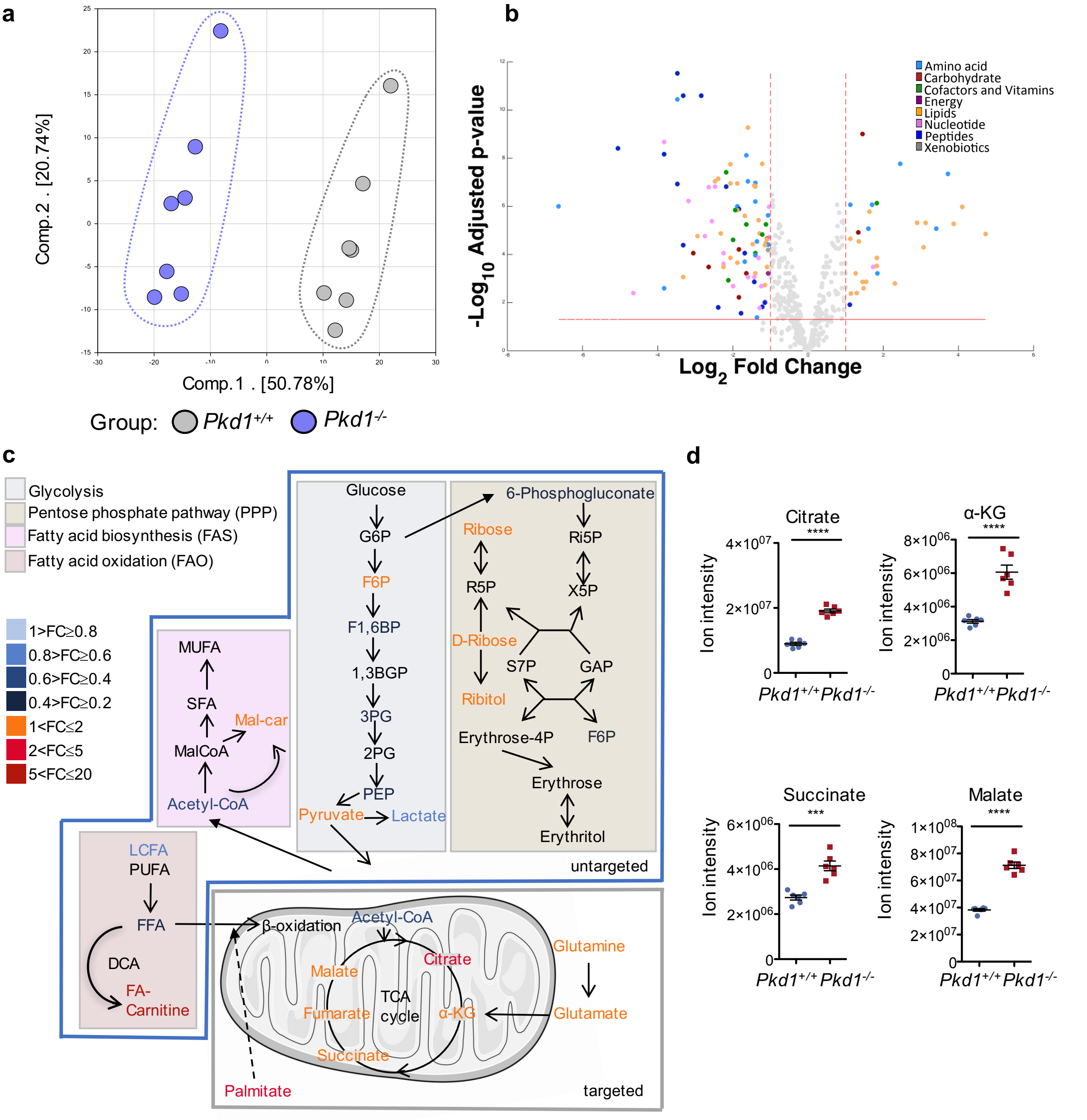

Supplementary Figure 2. a Principal component analysis (PCA) applied to the metabolites identified showed good separation between *Pkd1*$^{+/+}$ and *Pkd1*$^{-/-}$ cells. **b** Volcano plot of the metabolites profiled from *Pkd1*$^{+/+}$ and *Pkd1*$^{-/-}$ cells. 122 metabolites exhibited significant changes (adjusted *P* value < 0.05, absolute fold change > 2) when comparing *Pkd1*$^{+/+}$ with *Pkd1*$^{-/-}$ cells. 31 metabolites exhibited a significant increase whilst 91 metabolites exhibited a significant decrease. Paired Student's *t*-tests were used to calculate statistical significance, and *P* values were corrected by using the Benjamini-Hochberg procedure. Differentially abundant metabolites of different categories have been individually colour coded. **c** Significant metabolites were individually colour coded according to the pathway classification. Scheme of the glycolysis, pentose phosphate pathway, fatty acid oxidation and fatty acid biosynthesis *Pkd1*$^{+/+}$ and *Pkd1*$^{-/-}$ cells. Colour corresponds to the fold changes between *Pkd1*$^{-/-}$ and *Pkd1*$^{+/+}$ cells, orange-red labelled ones correspond to the metabolites more abundant, whereas blue labelled ones correspond to the metabolites less abundant in *Pkd1*$^{+/+}$ compared *Pkd1*$^{-/-}$ cells. Untargeted methodology is shown in the blue box whereas the targeted are shown in the grey box. **d** Levels of TCA intermediates citrate, α-KG, succinate and malate were assessed by LC-MS in *Pkd1*$^{+/+}$ and *Pkd1*$^{-/-}$ MEFs showing that they are significantly higher more abundant in the mutant cells compared to the controls. Dot plots showing means, SEM. *n.s.*, not significant ($P \geq 0.05$), * $P < 0.05$; ** $P < 0.001$;*** $P < 0.0001$;**** $P < 0.00001$. *t*-test for **d** and six technical replicates for **d**. All graphs represent the data of seven technical replicates of one experiment.

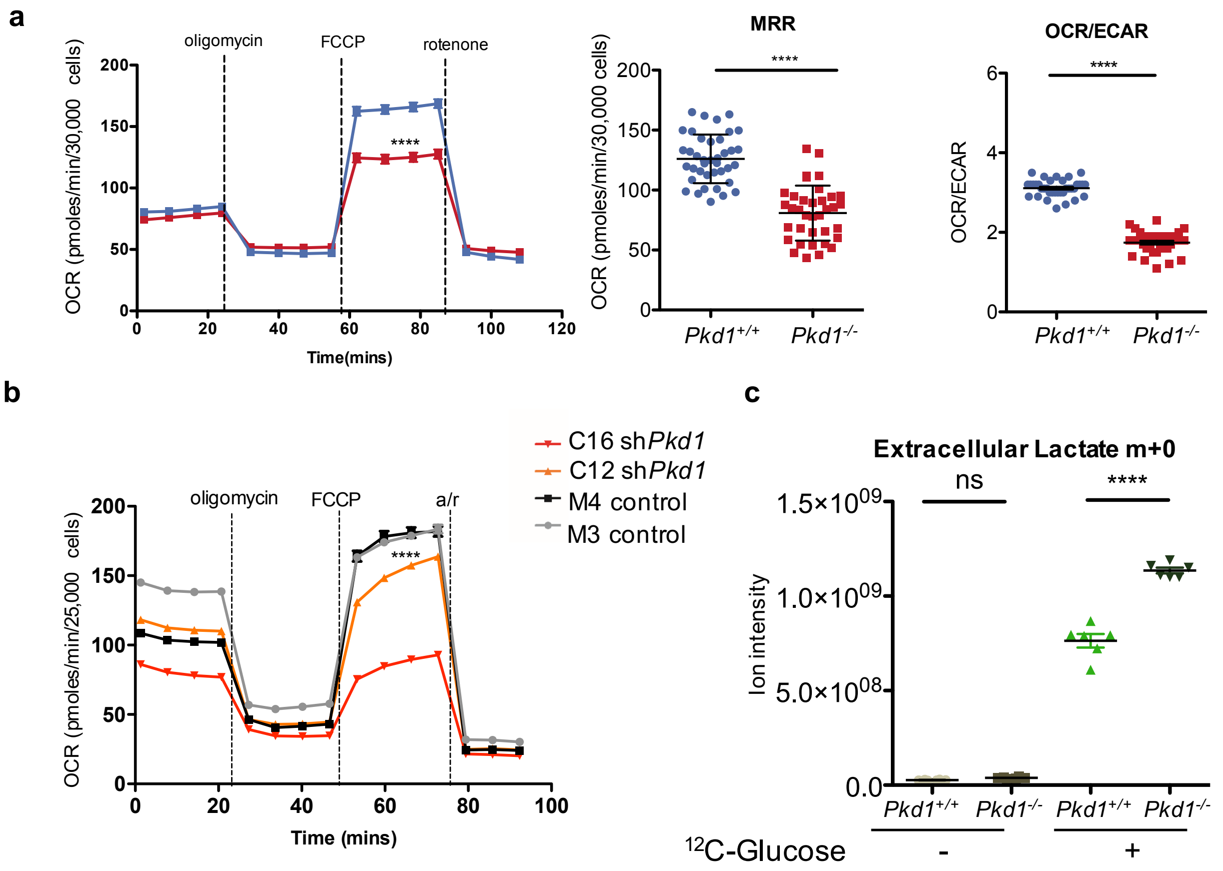

Supplementary Figure 3. a OCR and ECAR were measured using the XF Extracellular Flux Analyzer (Seahorse Bioscience) on primary mouse embryonic fibroblasts isolated from *Pkd1*$^{+/+}$ or *Pkd1*$^{\Delta C/\Delta C}$ embryos at E11.5. Dot plots showing means, MRR calculated from the 12th measurement (after subtraction of the 15th measurement), OCR/ECAR from basal measurement, 2nd time point. **b** sh*Pkd1* (C16, C12) cells compared to scrambled controls (M3, M4). **c** Extracellular lactate. Levels of unlabelled M+0 extracellular lactate were measured in *Pkd1*$^{-/-}$ and *Pkd1*$^{+/+}$ cells in the presence (+) or in the absence (-) of ^{12}C-glucose. Mean +/- SEM were indicated, n.s., not significant ($p \geq 0.05$), *$p<0.05$; **$p<0.01$;***$p<0.001$;****$p<0.0001$. *t*-test for **a** and **b**, ANOVA for **c**. Representative graphs of at least two independent experiments made from 33 to 38 (**a**) and from 20 (**b**) replicate wells. One experiment made with six technical replicates is represented in **c**.

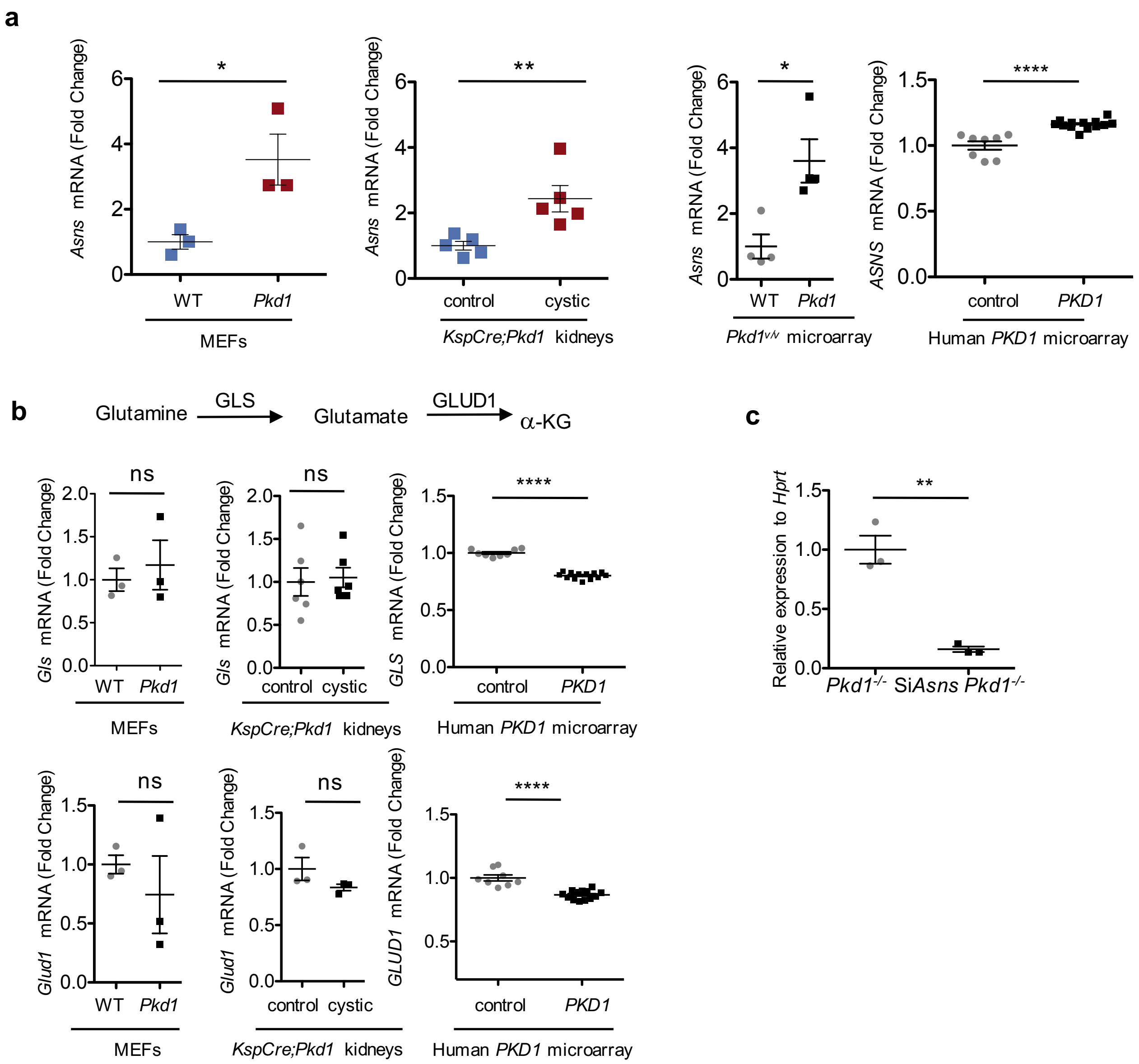

Supplementary Figure 4. a Dot plots showing means fold change, SEM, mRNA levels of *Asns* in $Pkd1^{-/-}$ compared to $Pkd1^{+/+}$ cells normalised to *Hprt*. *n*=3, three technical replicates of at least 3 independent experiments. Dot plots showing means fold change, SEM, mRNA *Asns* levels measured in control compared to cystic kidneys normalised to *Hprt*. *n*=5, two technical replicates of at least 2 independent experiments. Microarrays from $Pkd1^{V/V}$ P10 kidneys and human-derived microarrays of PKD patients samples showing upregulation of *ASNS*. **b** Quantitative RT-PCR of the genes coding for GLS or GLUD1, two key enzymes involved in glutamine usage (top scheme) normalised to *Hprt* shows that they are not increased in $Pkd1^{-/-}$ MEFs, cystic kidneys and human-derived microarray of PKD patients compared to the relative controls. **c** mRNA levels of *Asns* in $Pkd1^{-/-}$ compared to $Pkd1^{-/-}$ (mock) cells. Graphs represents. Mean +/- SEM were indicated, *n.s.*, not significant ($P \geq 0.05$), * $P<0.05$; ** $P<0.001$;*** $P<0.0001$;**** $P<0.00001$. *t*-test used for (**a**, **b** and **c**). (**a** and **b**) four independent biological replicates for $Pkd1^{V/V}$ animal model, eight (normal and minimal cyst) and 13 independent biological replicates for large, medium, small cyst for human *PKD1* microarrays (**a** and **b**). Graphs in **b** corresponding to the MEFs are representative of means of data of two independent experiments made on three experimental replicates and for the mice they are representative of five independent biological replicates (**b**) and three independent biological replicates (**c**).

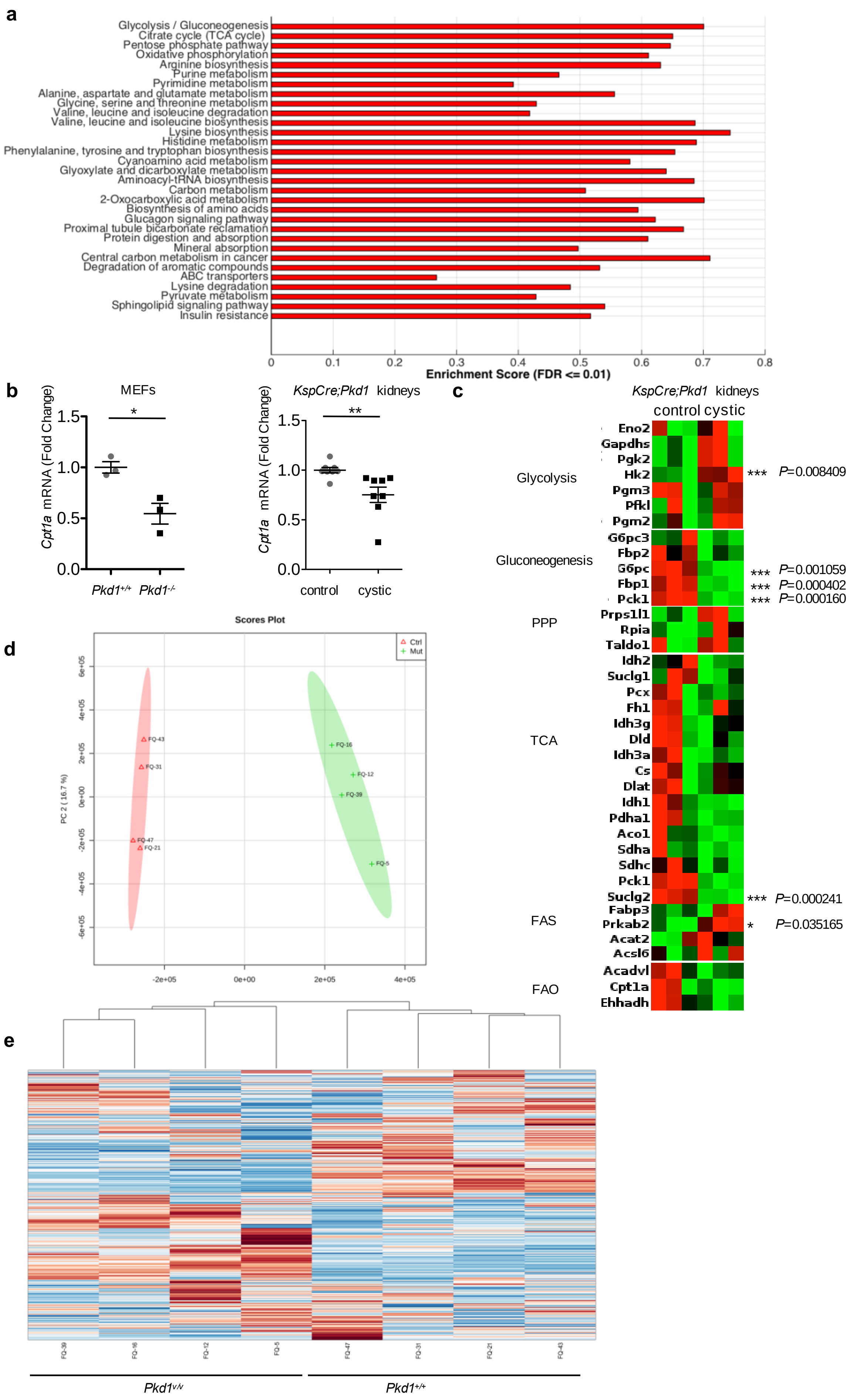
a
Glycolysis / Gluconeogenesis
Citrate cycle (TCA cycle)
Pentose phosphate pathway
Oxidative phosphorylation
Arginine biosynthesis
Purine metabolism
Pyrimidine metabolism
Alanine, aspartate and glutamate metabolism
Glycine, serine and threonine metabolism
Valine, leucine and isoleucine degradation
Valine, leucine and isoleucine biosynthesis
Lysine biosynthesis
Histidine metabolism
Phenylalanine, tyrosine and tryptophan biosynthesis
Cyanoamino acid metabolism
Glyoxylate and dicarboxylate metabolism
Aminoacyl-tRNA biosynthesis
Carbon metabolism
2-Oxocarboxylic acid metabolism
Biosynthesis of amino acids
Glucagon signaling pathway
Proximal tubule bicarbonate reclamation
Protein digestion and absorption
Mineral absorption
Central carbon metabolism in cancer
Degradation of aromatic compounds
ABC transporters
Lysine degradation
Pyruvate metabolism
Sphingolipid signaling pathway
Insulin resistance
0 0.1 0.2 0.3 0.4 0.5 0.6 0.7 0.8
Enrichment Score (FDR <= 0.01)
b
MEFs
Cpt1a mRNA (Fold Change)
1.5 1.0 0.5 0.0
*
Pkd1+/+ Pkd1-/-
KspCre;Pkd1 kidneys
Cpt1a mRNA (Fold Change)
1.5 1.0 0.5 0.0
**
control cystic
c
KspCre;Pkd1 kidneys
control cystic
Glycolysis
Eno2
Gapdhs
Pgk2
Hk2
Pgm3
Pfkl
Pgm2
*** P=0.008409
Gluconeogenesis
G6pc3
Fbp2
G6pc
Fbp1
Pck1
*** P=0.001059
*** P=0.000402
*** P=0.000160
PPP
Prps1l1
Rpia
Taldo1
TCA
Idh2
Suclg1
Pcx
Fh1
Idh3g
Dld
Idh3a
Cs
Dlat
Idh1
Pdha1
Aco1
Sdha
Sdhc
Pck1
Suclg2
*** P=0.000241
FAS
Fabp3
Prkab2
Acat2
Acsl6
* P=0.035165
FAO
Acadvl
Cpt1a
Ehhadh
d
Scores Plot
Ctrl
Mut
FQ-43
FQ-31
FQ-47
FQ-21
FQ-16
FQ-12
FQ-39
FQ-5
PC 2 (16.7 %)
6e+05 4e+05 2e+05 0e+00 -2e+05 -4e+05 -6e+05
-2e+05 0e+00 2e+05 4e+05
e
FQ-39 FQ-16 FQ-12 FQ-5 FQ-47 FQ-31 FQ-21 FQ-43
Pkd1v/v
Pkd1+/+

Supplementary Figure 5. a KEGG-Pathways Based Enrichment Analysis applied to the list of metabolites deriving from *in silico* simulation of increased glycolysis ranked according to the DFA algorithm. Only human pathways for which at least five metabolites are present in our mathematical model have been considered. Picture shows that 31 pathways result in an enrichment scores having FDR≤0.01, where FDR has been computed by applying Benjamini-Hochberg procedure. **b** Quantitative RT-PCR of genes encoding *Cpt1a* show decreased in *Pkd1*$^{-/-}$, MEFs and cystic kidneys compared to controls. **c** Clustergram performed non-supervised hierarchical clustering of the entire dataset displays a heat map with co-regulated genes across groups (cystic Vs. controls) for Glycolysis, Gluconeogenesis, PPP, TCA, FAS and FAO. The *P* values are calculated based on a Student's t-test of the replicate 2^ (- Delta C_T) values for each gene in the control group and treatment groups, and *P* values less than 0.05 are indicated in red. **d** Principal component analysis applied to the genes in microarrays data from *Pkd1*$^{V/V}$ animal model at P10 shows good separation between mutant versus control. **e** Hierarchical clustering analysis applied to the genes in microarrays data from *Pkd1*$^{V/V}$ animal model at P10 shows separation between mutant versus control. Graphs in **b** are representative of means of data of three independent experiments made on three experimental (MEFs) or biological replicates (Mice).

Supplementary Table 1: Primer Sequences

	forward	reverse
Epo	CATCTGCGACAGTCGAGTTCTG	CACAACCCATCGTGACATTTTC
Vefg	CTGTGCAGGCTGCTGTAACG	GTTCCCGAAACCCTGAGGAG
Fasn	TTGCTGGCACTACAGAATGC	AACAGCCTCAGAGCGACAAT
Cpt1a	AGTGGCCTCACAGACTCCAG	GCCCATGTTGTACAGCTTCC
Glud1	CCCAACTTCTTCAAGATGGTGG	AGAGGCTCAACACATGGTTGC
Gls	TTCGCCCTCGGAGATCCTAC	CCAAGCTAGGTAACAGACCCT
Asns	GGTTTTCTCGATGCCTCCTT	TGTGGCTCTGTTACAATGGTG
Hprt	TTATGTCCCCCGTTGACTGA	ACATTGTGGCCCTCTGTGTG

Supplementary Table 2: Abbreviations

Abbreviations:

α-KG, alpha-ketoglutarate
1,3BPG, 1,3-bisphosphoglycerate
BUN, blood urea nitrogen
DCA, dicarboxylic acids
FA-Carn, fatty acyl-carnitine
FAS, fatty acid synthesis
FAO, fatty acid oxidation
FI, Fold Induction
FFA, free fatty acids
F6P, fructose 6-phosphate
F1,6BP, fructose 1,6-bisphosphate
G6P, glucose 6-phosphate
GAP, glyceraldehyde 3-phosphate
Gln, glutamime
Glu, glucose
LCFA, long chain fatty acids
LC-MS, liquid chromatography coupled to mass spectrometry
MalCoA, malonyl-CoA
Mal, Malonate
MS, mass spectometry
MUFA, monounsaturated fatty acids
OAA, oxoloacetate
OCR, oxygen consumption rate
OxPhos, oxidative phosphorylation
PEP, phosphoenolpyruvate
6PG, 6-phosphpogluconate
2PG, 2-phosphoglycerate
3PG, 3-phosphoglycerate
PPP, pentose phosphate pathway
PUFA, polyunsaturated fatty acid
R5P, ribose 5-phosphate
Ri5P, ribulose 5-phosphate
SFA, saturated fatty acids
SPF, specific pathogen free
S7P, sedoheptulose 7-phosphate
X5P, xylulose 5-phosphate

Supplementary Methods

Blood Urea Nitrogen (BUN)

Analysis of serum samples was assessed by ILab Aries, a bench-top analyzer that can perform photometry, turbidimetry, and potentiometry tests (Instrumentation Laboratory, Werfen Group, Milan, Italy). BUN was detected using kits and controls supplied by ILab Aries. Standard controls were run before each determination to monitor the precision throughout the experiment, and the values obtained for controls were always within the expected ranges.

Immunohistochemistry

For immunohistochemistry (IHC), formalin-fixed paraffin-embedded consecutive sections (4 μm) were dewaxed and hydrated through graded decrease alcohol series. Stained for anti CD45 antibody (BD Biosciences) and anti-Collagen IV Antibody (abcam) as per manufacturer instructions. Antigen unmasking with Citrate Buffer pH 6.0, any endogenous peroxidase activity was quenched with 3% peroxidase water for 20 min at Room Temperature (RT). Antibodies dilution of 1:400, 1hr RT, and developed with Rat on Mouse HRP-Polymer (Biocare Medical,) or EXPOSE Rabbit specific HRP/DAB detection IHC kit (abcam). Immunostaining, DAB substrate chromogen was applied to sections for 5 min at RT and counterstained with Mayer's hematoxylin, dehydrated and mounted with Eukitt (BioOptica). External positive and negative controls were run simultaneously. Images were acquired using a Zeiss AxioImager M2m with AxioCam MRc5.

PCR Arrays

RNA was isolated from snap-frozen kidneys using RNeasy Plus Mini kit (Qiagen) following the manufacturer's instructions. In total, 1μg total RNA was subjected to single-stranded cDNA using the RT2 first strand kit (QIAGEN). The cDNA was used in the Mouse Glucose Metabolism (PAMM-006ZF) and Fatty Acid Metabolism (PAMM-007Z) RT^2 Profiler PCR arrays according to the manufacturer's instructions and assay on a LightCycler 480 Instrument (Roche). Data were analysed using the RT^2 Profiler program supplied by QIAGEN and normalized to the geometric mean of the housekeeping genes, actin-beta, glyceraldehyde-3-phosphate dehydrogenase, and heat shock protein 90kda for PAMM-006ZF and beta-actin and heat shock protein for PAMM-007Z.
