# Supplementary material for: Dissection of metabolic reprogramming in polycystic kidney disease reveals coordinated rewiring of bioenergetic pathways
Source: Commun Biol. 2018 Nov 16;1:194. doi: 10.1038/s42003-018-0200-x (PMC6240072; doi:10.1038/s42003-018-0200-x)
Supplement: Supplementary file 2 — Description of Supplementary Data [file 42003_2018_200_MOESM2_ESM.docx]

Descriptions of Additional Supplementary Information

**File Name:** Supplementary Data 1

**Description:** Statistical analysis for the 550 metabolites detected from mice kidneys. (Sheet1): fold change and statistical significance corrected for multiple comparison of the 550 metabolites detected by using Ultrahigh Performance Liquid Chromatography-Tandem Mass Spectroscopy (UPLC-MS/MS). (Sheet2): data for metabolites. Each compound is rescaled to set the median equal to 1.

**File Name:** Supplementary Data 2

**Description:** Statistical analysis for the 505 metabolites detected from MEF. (Sheet1):  fold change and statistical significance corrected for multiple comparison of 505 detected metabolites. (Sheet2): data for metabolites. Values for each sample were normalized by Bradford protein concentration. Each compound was then rescaled to set the median equal to 1.

**File Name:** Supplementary Data 3

**Description:** Lipidomics Table: Raw data of species molpercent data, Features are normalised to total lipid content in each sample. aov_pval indicates the p-value of an ANOVA based on the replicates for each condition.

**File Name:** Supplementary Data 4

**Description:** Metabolites and reactions whose concentrations are affected by *in silico* simulation of increased glycolysis. (Sheet1): reactions ranked according to DFA analysis. Reactions are ranked according to predicted mean absolute change of flux in the objective functions. In particular. Column 1: reactions; column 2: enzymes and transporters associated with each reaction; column 3: the average value of flux across the metabolic objectives according to DFA; column 4: the absolute value of column 3 (the value used to rank the reactions); columns 5 and 6: reactions direction in WT and increased glycolysis conditions, respectively. In particular, if 0 the reaction is not active, if 1 the reaction direction goes from left to right, and if -1 the reaction direction goes from right to left. (Sheet2): compounds ranked considering model compartments. Metabolites are ranked according to the predicted absolute change, as computed by DFA. (Sheet3): compounds ranked as in sheet2 without considering compartments and removing small molecules and cofactors.

**File Name:** Supplementary Data 5

**Description:** Subset of the *in silico* model reactions used to evaluate the change of metabolic pathways depicted in Figures 6A and 6B. Column 1: reactions; column 2: enzymes and transporters.

**File Name:** Supplementary Data 6

**Description:** Glucose metabolism, qPCR arrays, gene table, AverageCt, Average Delta (Ct), 2^(-Average(Delta(Ct)), Fold Change, p-value, Fold Regulation. Mean of 3 control and 3 cystic kidneys.

**File Name:** Supplementary Data 7

**Description:** Fatty acid metabolism, qPCR arrays, gene table, AverageCt, Average Delta (Ct), 2^(-Average(Delta(Ct)), Fold Change, p-value, Fold Regulation. Mean of 3 control and 3 cystic kidneys.

**File Name:** Supplementary Data 8

**Description:** *Pkd1*^V/V^ microarrays data. Samples code: a) Mutant: FQ-5, FQ-12, FQ-16, FQ-36; b) Control:  FQ-21, FQ-31, FQ-43, FQ-47.

**File Name:** Supplementary Data 9

**Description:** List of genes belonging to glycolysis, pentose phosphate pathway, TCA cycle/OXPHOS, fatty acid synthesis and fatty acid oxidation.

**File Name:** Supplementary Data 10

**Description:** Raw data of figures: 1,2,3,4,5 and raw data of supplemental figures of:1b, 1c, 2d, 3a, 3b, 3c, 4a,4b,4c and 5b.

**File Name:** Supplementary Data 11

**Description:** Raw data of tracing experiments.
